# Integrating the environmental and genetic architectures of aging and mortality

**DOI:** 10.1038/s41591-024-03483-9

**Published:** 2025-02-19

**Authors:** M. Austin Argentieri, Najaf Amin, Alejo J. Nevado-Holgado, William Sproviero, Jennifer A. Collister, Sarai M. Keestra, Midas M. Kuilman, Bigina N. R. Ginos, Mohsen Ghanbari, Aiden Doherty, David J. Hunter, Alexandra Alvergne, Cornelia M. van Duijn

**Affiliations:** 1 Nuffield Department of Population Health, University of Oxford, Oxford, UK; 2 Analytic and Translational Genetics Unit, Massachusetts General Hospital, Boston, MA USA; 3 Program in Medical and Population Genetics, Broad Institute of MIT and Harvard, Boston, MA USA; 4 Department of Psychiatry, University of Oxford, Oxford, UK; 5 Department of Epidemiology and Data Science, Amsterdam UMC, University of Amsterdam, Amsterdam, The Netherlands; 6 Amsterdam Reproduction and Development, Amsterdam UMC, University of Amsterdam, Amsterdam, The Netherlands; 7 Department of Epidemiology, Erasmus MC, University Medical Center Rotterdam, Rotterdam, The Netherlands; 8 Department of Epidemiology, Harvard TH Chan School of Public Health, Boston, MA, USA; 9 ISEM, University of Montpellier, CNRS, IRD, Montpellier, France

**Keywords:** Risk factors, Epidemiology, Predictive markers, Metabolic disorders, Diagnosis

## Abstract

Both environmental exposures and genetics are known to play important roles in shaping human aging. Here we aimed to quantify the relative contributions of environment (referred to as the exposome) and genetics to aging and premature mortality. To systematically identify environmental exposures associated with aging in the UK Biobank, we first conducted an exposome-wide analysis of all-cause mortality (*n* = 492,567) and then assessed the associations of these exposures with a proteomic age clock (*n* = 45,441), identifying 25 independent exposures associated with mortality and proteomic aging. These exposures were also associated with incident age-related multimorbidity, aging biomarkers and major disease risk factors. Compared with information on age and sex, polygenic risk scores for 22 major diseases explained less than 2 percentage points of additional mortality variation, whereas the exposome explained an additional 17 percentage points. Polygenic risk explained a greater proportion of variation (10.3–26.2%) compared with the exposome for incidence of dementias and breast, prostate and colorectal cancers, whereas the exposome explained a greater proportion of variation (5.5–49.4%) compared with polygenic risk for incidence of diseases of the lung, heart and liver. Our findings provide a comprehensive map of the contributions of environment and genetics to mortality and incidence of common age-related diseases, suggesting that the exposome shapes distinct patterns of disease and mortality risk, irrespective of polygenic disease risk.

## Abstract (web summary)

Based on a systematic analysis of environmental exposures associated with aging and mortality in the UK Biobank, the relative contributions of such exposures and genetic risk for mortality and a range of age-related diseases were compared, highlighting the potential beneficial effects of environment-focused interventions.

## Main

Human aging is a complex process that initially manifests as subclinical and biological changes that begin to accumulate from mid-life onward^1–3^. These systemic biological changes are major drivers of common age-related diseases^4–6^ and multimorbidity^7,8^, which in turn are the major causes of premature mortality worldwide^9^. While there have been major advancements in understanding the complex genetic etiology of age-related diseases, genetic studies show only a modest effect of the genome on lifespan^10,11^. Instead, a strong argument that nongenetic environmental factors play a key role in aging and premature mortality comes from the observation that global human lifespan has increased nearly twofold during the past 200 years, while the human genome is expected to have been stable in such a short period^12,13^. Epidemiological research has made major progress in relating individual environmental and behavioral exposures to age-related diseases and mortality, yet few studies have comprehensively examined the exposome (that is, the total set of interrelated environmental exposures throughout the life course) in relation to these outcomes^14,15^. In the field of genetic epidemiology, the use of genome-wide approaches has greatly increased the positive predictive value^16^ and reproducibility^17^ of findings, in particular for genetic variants conveying small effects on risk of the outcomes. Although individual genetic variants themselves convey a small increase in risk, aggregating these small effects over the genome shows that their joint effect can be substantial for various complex diseases. Exposome-wide study designs may provide similar advancements in the field of epidemiology.

It has been proposed that exposome-wide designs could provide crucial and systematic insights into the role of environmental exposures on aging^15^. While numerous environmental exposures have been previously associated with risk of mortality or with rates of biological aging in studies focused on smaller sets of exposures, so far no large-scale studies have used exposome-wide designs that can account for the correlation structure across the exposome to comprehensively identify exposures that have independent associations with both aging biology and population-level mortality and age-related disease rates. Further, the recent development of proteomic-based biological age clocks provide the opportunity to accurately characterize and measure signatures of aging biology using omics data^18^. While these proteomic age clocks are highly predictive of mortality and incident risk of most major chronic diseases^19,20^, there has been no exposome-wide study published so far that systematically identifies environmental exposures associated with aging biology.

**Fig. 1:**
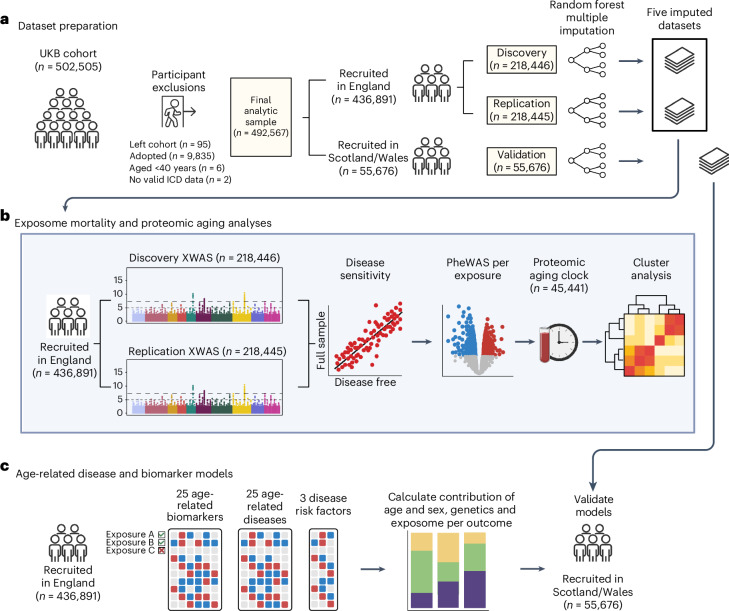
Study overview. **a**, After participant exclusions, UKB participants were split into independent discovery, replication and validation sets. Missing values were imputed separately within each group using random forest multiple imputation, resulting in five imputed datasets for each dataset. **b**, Among UKB participants recruited in England (*n* = 436,891), an exposome-wide association study (XWAS) for all-cause mortality was conducted using the discovery and replication sets. The discovery and replication sets were then pooled, and further analyses were conducted in the full sample to identify and remove replicated exposures that were sensitive to reverse causation (disease sensitivity) and mismeasurement (PheWAS per exposure). The remaining exposures were then tested cross-sectionally for associations with a previously developed proteomic aging clock (*n* = 45,441). We then conducted a final sensitivity analysis in the participants recruited in England (*n* = 436,891) to remove exposures sensitive to correlation bias (cluster analysis). **c**, Exposures surviving all analyses in **b** were then tested in relation to 25 age-related biomarkers, 25 age-related diseases and 3 common disease risk factors (hypertension, obesity and dyslipidemia). For mortality and each age-related disease, the relative contributions of age and sex, polygenic risk and exposome were calculated via multivariable Cox proportional hazard models. Multivariable models were validated in participants recruited in Scotland or Wales (*n* = 55,676), who were held out from all other analyses. Figure created with BioRender.com.

To address these gaps in the literature, we aimed to determine the contribution of the exposome to premature mortality and major age-related diseases, compared with the contribution of the genome. We developed a robust pipeline to address reverse causation and residual confounding (Fig. 1 shows a summary of the study design). We started by conducting an exposome-wide analysis using data from the UK Biobank (UKB; *n* = 492,567) to systematically identify exposures that are independently associated with risk of premature mortality and thus determine life expectancy. We then conducted a phenome-wide analysis for each mortality-associated exposure to remove exposures sensitive to confounding and mismeasurement. To determine whether these exposures contribute to the aging process instead of merely predicting death, we further limited exposures to those that are associated with a proteomic aging clock that we recently developed in a subset of UKB participants (*n* = 45,441)^19^. To overcome the strong correlation between exposures, we developed an approach to decompose confounding through hierarchical clustering of exposures. Finally, we assessed the effect of the identified environmental exposures in relation to (1) the incidence of 25 major age-related diseases, which are either major causes of death or highly prevalent in aging populations; (2) patterns of 25 biochemical markers for aging and morbidity; and (3) prevalence of three major risk factors for various common age-related disorders (obesity, hypertension and dyslipidemia). We also quantified the relative contribution of the exposome versus the genome in explaining variation in mortality and age-related diseases.

## Results

### Mortality and age-related disease rates

**Extended Data Fig. 1:**
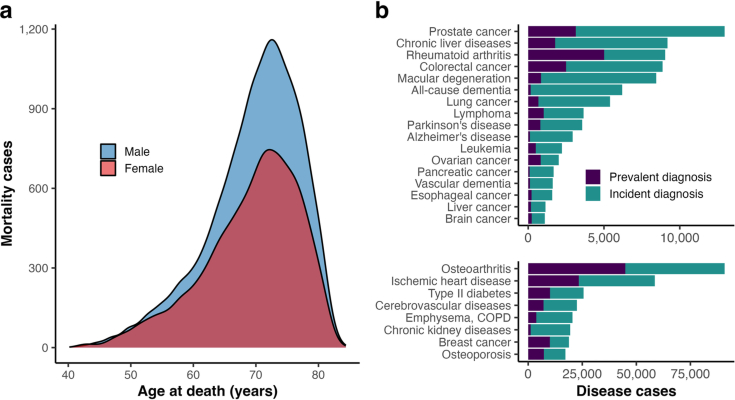
Mortality and disease incidence rates among UK Biobank participants. (**a**) The number of deaths in females and males according to age at death (in years) among UK Biobank participants who died during follow up (n = 31,716). (**b**) Numbers of prevalent and incident cases for all age-related diseases studied among UK Biobank participants recruited in England (n = 436,891). Note that diseases are put into two groups with different x-axis scales, since some diseases had far more cases than others.

This study included 492,567 UKB participants (Fig. 1). All analyses were carried out using UKB participants recruited in England (*n* = 436,891). Participants recruited in Scotland/Wales (*n* = 55,676) were held out as a validation set used only to validate final multivariable disease models. There were 31,716 deaths from all causes among participants recruited in England after a median 12.5 years of follow-up (Table 1). The majority (74.5%) of deaths were premature deaths (that is, occurring before 75 years of age; Extended Data Fig. 1a). Women had a lower all-cause mortality rate compared with men (5.4% in women versus 9.4% in men; Table 1). Mortality by cause of death for all participants is given in Supplementary Tables 3 and 4. Key demographic descriptive statistics for participants recruited in England are presented in Table 1. Baseline descriptive statistics are provided in Supplementary Table 1 for UKB participants with no prevalent disease used in sensitivity analyses and Supplementary Table 2 for UKB participants recruited in Scotland/Wales for validation analyses. The number of incident cases for the common age-related diseases studied in participants recruited in England ranged from 856 (brain cancer) to 45,879 (osteoarthritis), as shown in Extended Data Fig. 1b and Supplementary Table 5. Summary statistics for all cross-sectional outcomes (3 common disease risk factors and 25 biochemical aging markers) are given in Supplementary Tables 5 and 6.

**Table 1:** Baseline descriptive statistics for UKB participants recruited in England

	Female (*N* = 237,634)	Male (*N* = 199,257)	Total (*N* = 436,891)
**Age**			
Mean (s.d.)	56 (8.0)	57 (8.2)	57 (8.1)
**Household income**			
Less than 18,000	52,139 (21.9%)	38,416 (19.3%)	90,555 (20.7%)
18,000–30,999	58,496 (24.6%)	45,827 (23.0%)	104,323 (23.9%)
31,000–51,999	52,229 (22.0%)	48,178 (24.2%)	100,407 (23.0%)
52,000–100,000	37,443 (15.8%)	39,514 (19.8%)	76,957 (17.6%)
Greater than 100,000	9,742 (4.1%)	10,884 (5.5%)	20,626 (4.7%)
**Education years**			
7 years	39,642 (16.7%)	33,716 (16.9%)	73,358 (16.8%)
10 years	46,951 (19.8%)	27,632 (13.9%)	74,583 (17.1%)
13 years	13,922 (5.9%)	10,134 (5.1%)	24,056 (5.5%)
15 years	31,779 (13.4%)	20,463 (10.3%)	52,242 (12.0%)
19 years	30,058 (12.6%)	38,388 (19.3%)	68,446 (15.7%)
20 years	72,867 (30.7%)	66,742 (33.5%)	139,609 (32.0%)
**Ethnicity**			
White	223,428 (94.0%)	187,256 (94.0%)	410,684 (94.0%)
Asian	5,172 (2.2%)	5,344 (2.7%)	10,516 (2.4%)
Black	4,452 (1.9%)	3,210 (1.6%)	7,662 (1.8%)
Mixed	1,610 (0.7%)	938 (0.5%)	2,548 (0.6%)
Other	2,388 (1.0%)	1,737 (0.9%)	4,125 (0.9%)
**BMI**			
Mean (s.d.)	27 (5.2)	28 (4.2)	27 (4.8)
**Smoking status**			
Never	141,414 (59.5%)	97,119 (48.7%)	238,533 (54.6%)
Previous	74,753 (31.5%)	77,122 (38.7%)	151,875 (34.8%)
Current	20,591 (8.7%)	24,223 (12.2%)	44,814 (10.3%)
**Home area population density**			
Urban	203,583 (85.7%)	171,299 (86.0%)	374,882 (85.8%)
Rural	34,051 (14.3%)	27,958 (14.0%)	62,009 (14.2%)
**Mortality**			
Alive	224,740 (94.6%)	180,435 (90.6%)	405,175 (92.7%)
Dead	12,894 (5.4%)	18,822 (9.4%)	31,716 (7.3%)

Mortality rates are for the 11- to 15-year study follow-up period. Descriptive statistics are calculated using the first imputed analysis dataset and are not pooled across imputed datasets. BMI, body mass index.

### Exposome-wide analysis of mortality

**Fig. 2:**
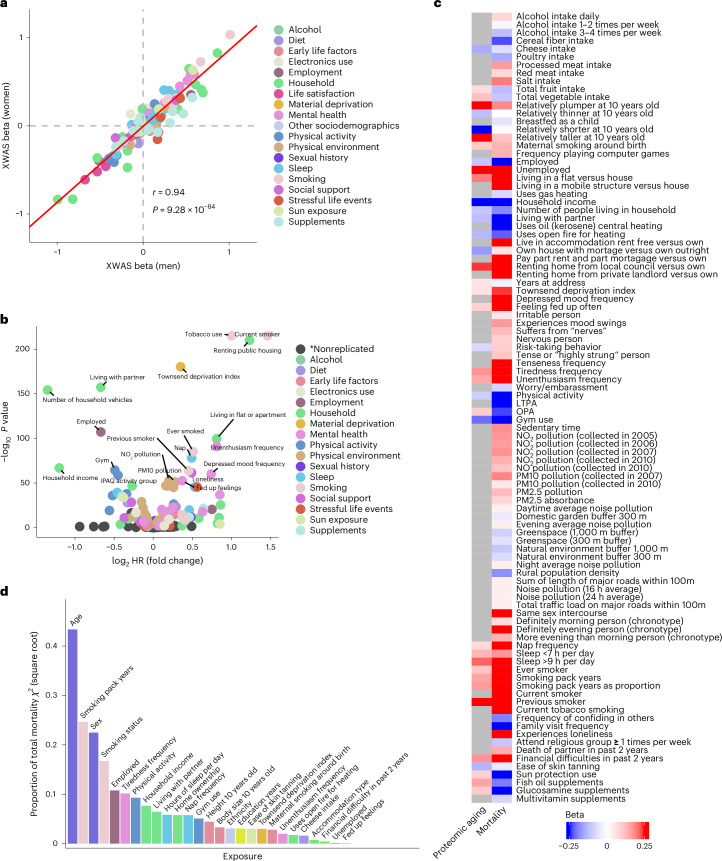
Environmental architecture of mortality in the UKB. **a**, The correlation (Pearson *r*) between regression coefficients (beta) for the association between each exposure and mortality calculated separately in women (*n* = 237,637) and men (*n* = 199,257). The *P* value for the significance of the Pearson correlation is also given. **b**, Volcano plot of log-transformed *P* values and fold change (calculated as log_2_ of the HR) for all XWAS associations for mortality in the final pooled analysis. Each point represents the effect and *P* value for the association between a single exposure and all-cause mortality from a Cox proportional hazard model in the XWAS discovery analysis (*n* = 218,446). Exposures that were FDR significant in both the discovery and replication stages are colored, whereas associations that were not replicated are indicated in dark gray and grouped in the category *nonreplicated. The top 20 points according to *P* value are labeled. **c**, A heat map of *β* coefficients representing associations between all exposures (only those passing disease and phenome-wide sensitivity analyses) and mortality (from the XWAS discovery analysis; *n* = 218,446) and proteomic aging (*n* = 45,441). **d**, Importance of individual exposures, as assessed by a multivariable model including age, sex and all 25 exposures associated with mortality and proteomic aging that passed all sensitivity analyses (*n* = 436,891). The importance of each variable was determined using a Wald test from ANOVA, and was calculated as the proportion of that variable’s Wald *Χ*^2^ relative to the total model *Χ*^2^. Note that the *y*-axis values were transformed by taking the square root to improve visualization. Physical activity was measured using the International Physical Activity Questionnaire (IPAQ). LTPA, leisure time physical activity; OPA, occupational physical activity; PM, particulate matter.

**Extended Data Fig. 2:**
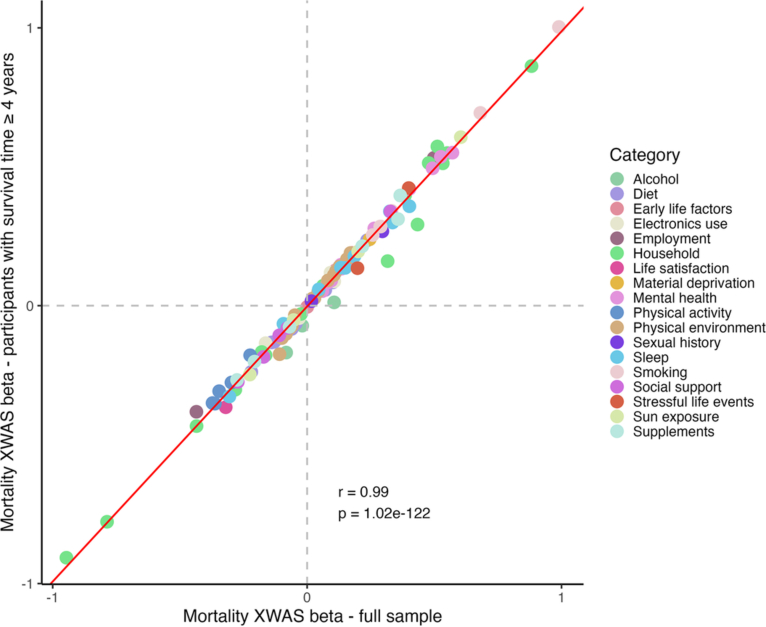
Mortality XWAS associations by different intervals of follow up time. Correlation between mortality XWAS regression estimates (betas) calculated in the full pooled sample (x-axis; n = 436,891) and the subset of participants excluding those who died within the first 4 years of follow up (y-axis; n = 431,394). Correlation between betas (Pearson r) is shown, as is the p-value for the correlation. A best fit line is fitted by regressing the betas from the y-axis onto the betas from the x-axis.

**Extended Data Fig. 3:**
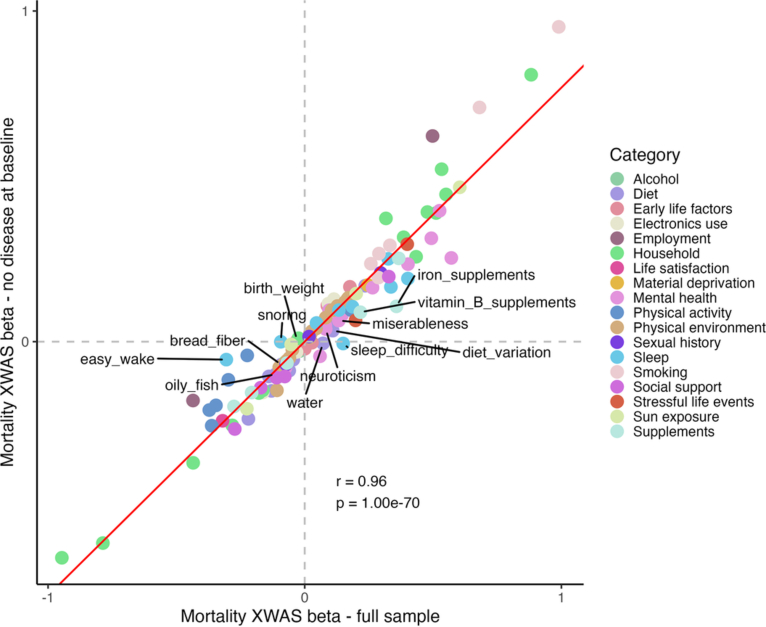
Mortality XWAS associations accounting for prevalent disease. Correlation between mortality XWAS regression estimates (betas) calculated in the full analytic sample (x-axis; n = 436,891) and the subset of participants with no disease or poor health at baseline (y-axis; n = 221,067). Correlation between betas (Pearson r) is shown, as is the p-value for the correlation. A best fit line is fitted by regressing the betas from the y-axis onto the betas from the x-axis. Labeled points are those variables that were flagged during the disease indicator interaction analysis.

Exposome-wide association study (XWAS) analyses of all-cause mortality were conducted by serially testing 164 environmental exposures in relation to mortality via Cox proportional hazards models in independent discovery and replication subsets of the UKB study population (Fig. 1). We limited our investigation of exposures to the external exposome only, meaning that internal biochemical responses to exposures were not included in our definition of the exposome. We further excluded exposures that reflect treatment for an already diagnosed disease, such as drug and medication use. No notable differences were observed in XWAS regression coefficients when these were calculated separately in females and males (Fig. 2a). In a final mortality XWAS combining females and males, 110/164 exposures (67.1%) were significantly replicated (Fig. 2b). Smoking, renting public housing (compared with home ownership) and Townsend deprivation index were the exposures most significantly associated with increased mortality risk. Living with a partner (compared with living alone or with other non-partners), the number of household vehicles, being employed and household income were the exposures most significantly associated with decreased mortality risk. We further conducted sensitivity analyses in which exposome-mortality associations were re-assessed by (1) excluding participants who died within the first 4 years of follow-up and thus may have already had disease at the assessment of the exposure (Extended Data Fig. 2) and (2) testing interactions between each exposure and a baseline poor health indicator (Extended Data Fig. 3). These led to the exclusion of 15 exposures whose associations with mortality were probably completely explained by prevalent disease status (Methods), leaving 95 remaining exposures. Summary statistics from all mortality XWAS analyses are given in Supplementary Files 3–7.

### Detecting residual confounding

For each of the 95 replicated exposures, we conducted a phenome-wide association study (PheWAS) where the exposure was treated as the outcome variable and regressed against all baseline phenotypes present in the UKB using either logistic or linear regression. We detected a further ten exposures that associated extremely strongly with either (1) disease, frailty or disability phenotypes, or (2) another exposure such that it probably does not represent independent information. For example, we found that one of the exposures most significantly associated with mortality in the XWAS, number of vehicles in a participant’s household (mortality hazard ratio (HR) 0.39, *P* = 5.2 × 10^−155^), was very strongly associated with greater household income (*β* *=* 1.1, *P* < 8.1 × 10^−12^), while inversely associated with living in council housing versus home ownership (*β* *=* −0.98, *P* < 5 × 10^−56^) and being unemployed due to a disability (*β* *=* −0.62, *P* < 1.4 × 10^−245^). These findings indicate that the association between the numbers of vehicles and mortality is probably explained by confounding from socioeconomic and disability status (Supplementary Fig. 1). All exposures showing evidence for residual confounding from PheWAS were discarded, leaving 85 remaining exposures. Summary statistics from all PheWAS are given in Supplementary Files 62–177.

### Identifying exposures involved in biological aging

Among the subset of UKB participants with plasma proteomics data collected at baseline (*n* = 45,441), we further tested the associations between each of the 85 remaining exposures and an established proteomic age clock^19^. This clock has been previously demonstrated to associate with mortality, 18 major chronic age-related diseases (including all the non-cancer diseases and four of the cancers studied here), multimorbidity and aging related phenotypes (for example, frailty index and cognitive function). It is therefore a suitable multidimensional measure of biological aging that has been demonstrated to capture aging biology relevant across the aging outcomes studied here. Specifically, we tested the association between each of the 85 remaining exposures and a proteomic age gap, which represents the difference (in years) between a participant’s protein-predicted age and calendar age. Exposures either not showing an association with proteomic aging or showing an association in the opposite direction from mortality were taken to indicate exposures that either do not have an impact on aging biology or that probably suffer from residual confounding. Of the 85 exposures tested, 57 exposures were discarded as either (1) not associated with proteomic aging after false discovery rate (FDR) correction or (2) associated with proteomic aging and mortality in opposite directions of effect. Exposures ruled out during this stage included some dietary exposures (intake of alcohol, meat, cereal fiber, salt, multivitamins and glucosamine supplements), mental health (depressed mood, mood swings, irritation and nervousness), air pollution and greenspace exposure, and certain social interactions (frequency of visiting family and friends or confiding in others and loneliness). This left 28 exposures significantly associated (FDR *P* value < 0.05) with both premature mortality and proteomic aging with an effect in the same direction for both outcomes (Fig. 2c). Summary statistics for proteomic aging analysis are given in Supplementary File 8.

### Dimension reduction and adjusting for correlation structure

**Fig. 3:**
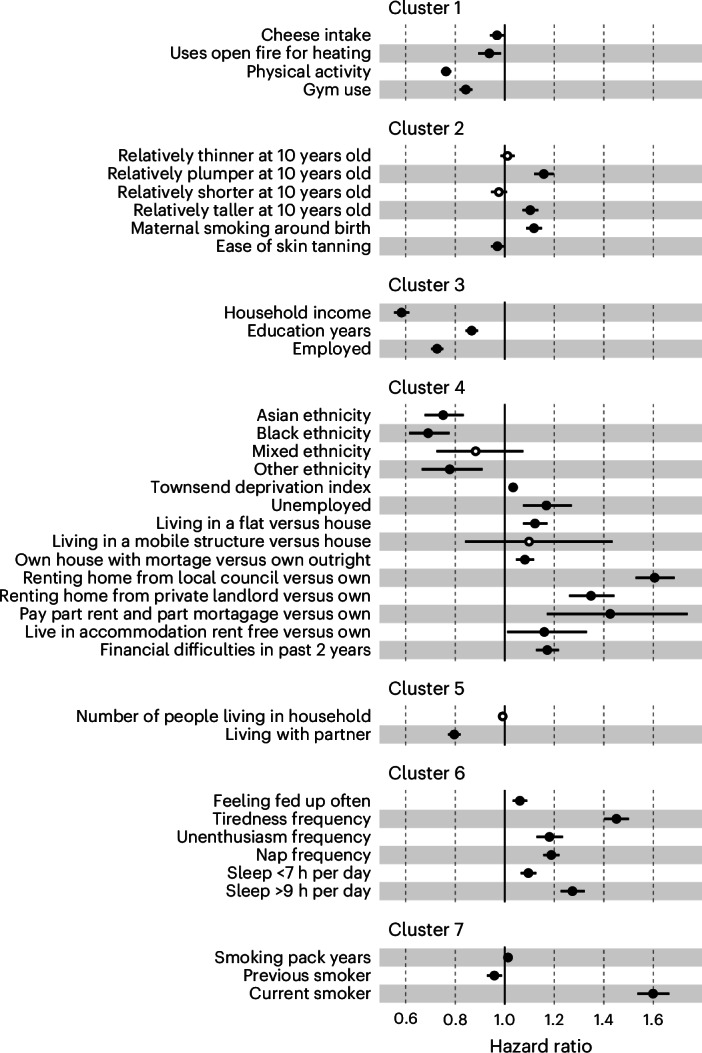
Forest plot of exposome associations with all-cause mortality (*n* = 436,891) in multivariable models for each individual cluster of correlated exposures. The models were Cox proportional hazard models calculated using age as the timescale, stratified by 5-year birth cohorts and sex, and adjusted for UKB assessment center, years of education, household income and ethnicity (only if the covariate was not already in the cluster model). The regression estimates are shown with 95% confidence intervals, and estimates not significant at *P* < 0.05 are shown as hollow points. The *P* values were not adjusted for multiple comparisons. Physical activity was measured via the IPAQ.

As expected, we observed high degrees of correlation between exposures replicated in the XWAS, with 90% of variable pairs showing evidence of significant correlation with a Bonferroni-corrected *P* value below 0.001. This indicated that some mortality associations observed in the XWAS may be confounded due to this correlation structure or multicollinearity. To address this, we used hierarchical clustering to organize the 28 exposures that were replicated in the XWAS and passed all sensitivity analyses detailed above into seven unique clusters. We then conducted multivariable mortality models within each cluster by adding all exposures from the cluster into a single Cox model. We discarded exposures that did not pass multicollinearity tests or were not significant in this within-cluster model. Using this method, we identified 25 exposures that were independently associated with mortality (Fig. 3).

Of these 25 exposures that were associated with proteomic aging and independently associated with mortality in the cluster multivariable analysis, only two were non-modifiable risk factors (Asian/Black/other ethnicity compared with white, being relatively taller at 10 years old compared with being of average height or shorter). The remaining 23 can be considered independent risk factors that are potentially modifiable. Among all significant exposures in the final cluster models, the largest protective effect sizes were found for household income; being employed; Asian, Black or other ethnicity (compared with white); self-reported physical activity (International Physical Activity Questionnaires (IPAQ)); and living with a partner (compared with living alone or with other nonpartners). All with HRs <0.8. The largest detrimental effect sizes were seen for current smokers, living in council housing versus home ownership and frequency of feeling tired (all with HRs >1.4).

### Patterns of multimorbidity and biological mechanisms

**Fig. 4:**
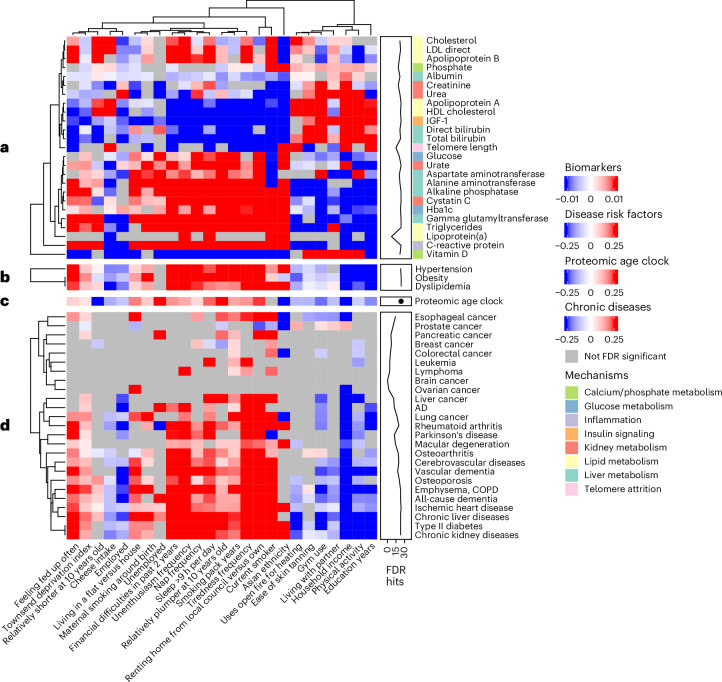
Environmental architectures of age-related biological mechanisms and diseases in the UKB. **a**, A heat map showing associations between each mortality-associated exposure and aging biomarkers. **b**, A heat map showing associations between each mortality-associated exposure and common disease risk factors. **c**, A heat map showing associations between each mortality-associated exposure and proteomic aging. **d**, A heat map showing associations between each mortality-associated exposure and age-related chronic diseases. The colors in the heat maps represent regression coefficients (*β*) for associations between exposures and biomarkers/diseases. A line annotation track is shown that counts the total number of FDR significant associations for each outcome. For the heat map in **a**, an additional annotation track shows the primary biological mechanism associated with each aging biomarker. For nominal categorical variables with more than one response level, the association for the level with the strongest *P* value is reported and the exposure’s label reflects the response category shown. COPD, chronic obstructive pulmonary disease; FDR, false discovery rate; HDL, high-density lipoprotein; IGF-1, insulin-like growth factor-1; LDL, low-density lipoprotein.

To test whether the 25 identified exposures were associated with development of age-related disease as part of the pathway to premature mortality, we tested each exposure individually in relation to incidence of 25 age-related diseases via Cox proportional hazards models (8–15 years of follow-up). We further tested each exposure individually in relation to patterns of 25 age-related biomarkers and three common disease risk factors (hypertension, obesity and dyslipidemia). Each of the 25 exposures was associated with a wide range of aging biomarkers that span diverse organ systems and mechanisms (Fig. 4a). On average, each exposure was associated with a total of 22 biomarkers (out of 25). Overall, two exposures were associated with all 25 biomarkers (smoking status and ethnicity), two with 24/25 biomarkers (hours of sleep and household income) and six with 23/25 biomarkers (frequency of feeling unenthusiastic, Townsend deprivation index, home ownership (compared with renting or living rent free), years of education, relatively plumper body size at 10 years old (compared with average or slimmer) and experiencing financial difficulty in the past 2 years). Metabolic risk factors for various common disorders (obesity, hypertension and dyslipidemia) were cross-sectionally associated with nearly every exposure studied (Fig. 4b). By design, all exposures were associated with proteomic aging (Fig. 4c).

Each of the 25 exposures was also associated with concurrent incidence of multiple age-related diseases (Fig. 4d), indicating that the exposome is a potential catalyst of disease multimorbidity. On average, each exposure was associated with a total of 15 age-related diseases (out of 25). Smoking (both current smoking status and pack years) was associated with 21 diseases. Household income, Townsend deprivation index, home ownership (compared with renting or living rent free) and frequency of feeling tired were associated with 19 diseases. Physical activity, hours of sleep, going to the gym and being relatively plumper at 10 years old (compared with average or slimmer) were associated with 17 diseases. Of note, we found no associations between any exposure and incidence of brain cancer. Summary statistics from all biomarker, age-related disease and common disease risk factor analyses are given in Supplementary Files 9–33.

We carried out additional sensitivity analyses to interrogate the observed association between current smoking and decreased risk of incident prostate cancer. This inverse association has been well documented in previous studies^21–23^, and it has been posited that those who do not smoke may be more likely to undergo a prostate-specific antigen test and receive a diagnosis, whereas those who smoke may be less likely to undergo testing and therefore would be undiagnosed or not diagnosed until a much later stage. However, after adjusting for and stratifying by prostate-specific antigen test status (Supplementary Methods), we found no change in the inverse association observed between smoking and prostate cancer (Supplementary Table 12).

### Environmental and genetic architectures of aging

To determine the contribution of age and sex, the exposome and genome in describing variation in premature mortality and the 25 studied age-related diseases, we calculated stepwise multivariable Cox models beginning with just age and sex (model 1), then adding either publicly available polygenic risk scores (PRS) as an approximation of genetic influence (model 2), or all independent exposures associated with the disease as an approximation of the exposome (model 3) and finally adding both the exposome and PRS together (model 4). The models were fitted among participants recruited in England (*n* = 436,891) and then validated in participants recruited in Scotland/Wales (*n* = 55,676).

**Fig. 5:**
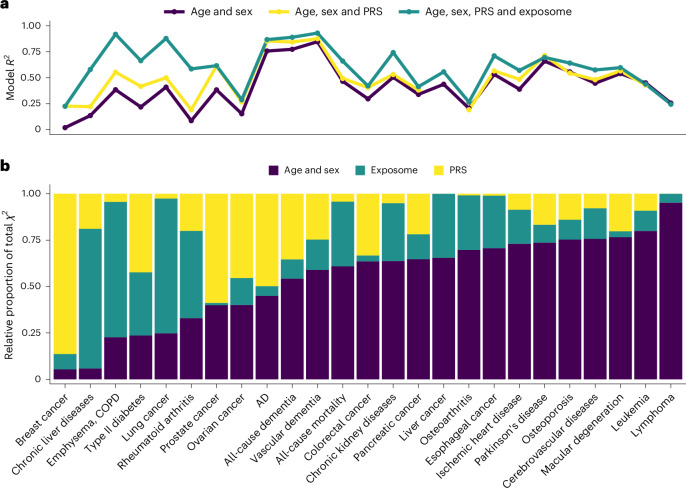
Combined environmental and genetic architectures of mortality and age-related diseases. **a**, A plot showing *R*^2^ values calculated across studied outcomes for several sequential multivariable models: model 1 containing age and sex (purple); model 2 containing age, sex and PRS (yellow); and model 4 containing age, sex, PRS and exposome (green). If a PRS was not available for a particular outcome, then the green *R*^2^ shows the results from model 3 (age, sex and exposome). The *R*^2^ values are shown from the validation analyses (*n* = 55,676). **b**, The variable importance for age, sex, polygenic risk and exposome for all outcomes studied in model 4 conducted among UKB participants recruited in England (*n* = 436,891). The importance of each variable was determined using a Wald test from ANOVA, and was calculated as the proportion of that variable’s Wald *Χ*^2^ relative to the total model *Χ*^2^ for each category so that they sum to 1. The total importance for PRS also includes the genetic principal components and genotyping batch covariates used. PRS used for mortality models include PRS for all other diseases and phenotypes shown (22 in total). Note that PRS information was not available for liver cancer or lymphoma and is not included in the models. Ovarian, breast and prostate cancer models were sex specific and sex was not included in model 4 for these outcomes. AD, Alzheimer’s disease; COPD, chronic obstructive pulmonary disease; PRS, polygenic risk score.

Starting with mortality, we observed that, compared with a model containing age and sex, adding the PRS for 22 diseases that are either major causes of death or common aging phenotypes only increased the total mortality model *R*^2^ by 2–3 percentage points (pp) (Fig. 5a and Extended Data Tables 1 and 2, model 2 versus 1). By contrast, we found that adding all 25 independent exposures associated with mortality (that is, exposome) to age and sex increased the total mortality model *R*^2^ by 16–19 pp (model 3 versus 1). Adding the 25 exposome variables to the model with age, sex and all PRS increased the total mortality model *R*^2^ by 14–17 pp (model 4 versus 2). However, adding PRS to a model already containing age, sex and the exposome barely increased the *R*^2^ by less than 1 pp (model 4 versus 3). While the combined effect of the exposome explained a large proportion of mortality variation, we found that individually most exposures only explained a small proportion of total mortality variation (Fig. 2d).

To test whether we underestimated the genetic influence on mortality and lifespan using this disease PRS approach, we also conducted a sensitivity analysis where we further included *APOE* genotype status (using variants rs429358 and rs7412) and a variant in *FOXO3* previously associated with longevity (rs2802292)^24^ in model 2 and model 4. We found that inclusion of these additional important aging and longevity genetic variants led to virtually no change in our mortality results (Supplementary Table 16). Further, to test whether the relative amount of variation in mortality explained by disease-related PRS was being diluted using all-cause mortality as the outcome, we conducted a sensitivity analysis in which we retested models 2–4 but with the outcome being mortality caused by any of the 25 chronic diseases studied here instead of all causes. We observed the *R*^2^ values for each model to be slightly improved compared with the corresponding all-cause mortality model. However, we still found that the exposome (model 3) explains approximately 14–16 pp greater variance in chronic disease-specific mortality compared with model 2 including all PRS together (Supplementary Tables 14 and 15). Furthermore, the addition of PRS on top of the exposome model only increased the *R*^2^ by approximately 1 pp (model 4 versus 3).

Models including age and sex, exposome and PRS (model 4) captured >50% of variation in most outcomes studied in the validation set, with the exception of colorectal cancer, pancreatic cancer, leukemia, breast and ovarian cancers, lymphoma and osteoarthritis (Fig. 5a and Extended Data Tables 1 and 2). For all-cause mortality and all age-related diseases studied, the relative importance of age, sex, exposome and PRS are shown in Fig. 5b according to the relative proportions of the total model chi-squared (*Χ*^2^) that each variable category explained in model 4. The exposome explained the most of disease variation for lung cancer, emphysema/chronic obstructive pulmonary disease (COPD), chronic liver diseases and rheumatoid arthritis. Certain outcomes seem to be more influenced by polygenic risk than the exposome, such as breast and prostate cancers, Alzheimer’s disease (AD), all-cause dementia, macular degeneration and colorectal cancer. Last, all-cause mortality as well as a number of disorders including esophageal cancer, ischemic heart disease and cerebrovascular diseases showed age and sex as the most influential determinants, but also showed that the exposome explained the majority of the residual variation not explained by age and sex.

As a final sensitivity analysis, we attempted to compare the explanatory power of the baseline self-reported physical activity variables used versus an objective measure of physical activity using accelerometer data available in a subset of UKB participants (*n* = 103,672; Supplementary Methods). Objectively collected total physical activity explained a greater amount of the mortality variation in our UKB sample by 3pp, indicating that our overall estimate of the variation of mortality explained by baseline self-reported physical activity measures underrepresents the total influence of objectively measured physical activity on mortality by only approximately 3pp (Supplementary Table 13).

## Discussion

Our study provides the first assessment of the relative contributions of environmental and genetic influences on aging. We identify 25 independent exposures associated with premature mortality, proteomic aging, biochemical markers of aging and age-related diseases. We find that the major drivers of premature death and aging in our sample are smoking, socioeconomic status and deprivation, ethnicity, physical activity, living with a partner, sleep and mental and physical wellness including tiredness, as well as early life exposures including height and body size at 10 years and maternal smoking around birth. Our study shows that the environmental architecture of mortality and aging is composed of many interrelated factors, which individually may sometimes only capture a small proportion of premature mortality variation but when combined additively explain a substantial amount of variation for premature mortality, far exceeding that of polygenic risk. We further demonstrate that the associations we observed among these 25 independent exposures and premature mortality risk and proteomic aging are probably not explained by reverse causation or residual confounding.

The 25 mortality-associated exposures we identify are associated with a common signature of proteomic aging, 24 major age-related diseases and their metabolic risk factors and multimorbidity. Our results demonstrate that many age-related diseases share a common environmental etiology that ultimately leads to premature mortality and thus shapes life expectancy. We observed high variability across disorders in the contribution of the genome and exposome. Certain disorders, such as several cancers (breast, ovarian, prostate and colorectal), AD, all-cause dementia and macular degeneration, were found to be predominantly influenced by polygenic risk (that is, the genome) rather than by the exposome, while others, such as cerebrovascular diseases, ischemic heart disease, COPD, rheumatoid arthritis and liver and kidney diseases showed age, sex and the exposome as the most influential determinants.

While numerous previous studies have documented the significant roles of physical activity, smoking, sleep and individual socioeconomic status (household income, employment and home ownership status) in shaping mortality risk^25,26^, we provide a more expanded picture of the myriad biological mechanisms and disease pathways associated with each. Although the key role of physical activity for maintaining a healthy body weight has long been recognized, its role in aging and life expectancy has been less clear as extreme physical activity may increase oxidative stress and thus increase aging^27^. The finding that being shorter at age 10 years is associated with reduced proteomic aging and lower risk of mortality is in line with the numerous studies suggesting that smaller animals within the same species have a higher life expectancy^28,29^. The finding that being relatively plumper at age 10 years and maternal smoking around birth have an impact on increased proteomic aging in adulthood and a higher risk of premature mortality supports the view that life course prevention of aging is key.

Overall, we found that a large number of mortality-associated exposures (66%) were not associated with proteomic aging. Of note, nearly all exposures related to self-reported diet (for example, intakes of cereal fiber, red meat, fruit and vegetables) and physical environment (for example, pollution and greenspace) were associated with mortality in the XWAS but were not associated with proteomic aging. Although the sample size for the proteomic aging study is smaller, we have previously shown that this proteomic age clock is a powerful predictor of major diseases^19^. It may be that while these exposures are strong determinants of mortality, they may suffer from reverse causation (that is, people change their diet after developing an illness) or the exposures themselves may not be strongly related to aging over the life course and work through other pathways. Alternatively, the lack of associations between self-reported dietary exposures and proteomic aging may reflect confounding or a lack of precision in these self-report measures^30^.

Our research indicates that risk of premature mortality is lower for Black, Asian and ‘other’ ethnicities compared with whites in the UKB, even after adjustment for a large suite of sociodemographic and deprivation factors. This mirrors previous research using national UK census and death registration data showing that life expectancy is lower for whites compared with all other ethnic groups in the UK^31^. However, these same non-white ethnic groups also tend to live in higher deprivation areas, report poorer self-rated health and report poorer experiences of using health services in the UK^32^. More research is required to understand the factors producing lower mortality risk for UK minorities despite higher levels of deprivation.

There are several limitations to note for our analysis. First, despite our prospective study design and careful evaluation of reverse causation and confounding, reported associations may not be causal. Although we observed consistent association patterns across different outcomes (mortality, proteomic aging, blood biochemistry biomarkers, common disease risk factors and incident diseases) and validated our findings in a holdout validation set of participants recruited in Scotland/Wales, causality will need to be formally established through appropriate study designs. Second, the UKB population is healthier and more affluent than the general UK population^33^, and the mortality trends in our population are also not representative of the general UK population mortality in terms of age at death. However, this plays out as a strength of our study since it allows us to identify exposures associated with premature mortality. Third, we could not capture exposome dynamics across the life course, since all exposures were only measured at one time point in the full cohort. We also have not captured all possible exposome influences, as we were limited to the exposures available in the UKB. Our estimates of the proportion of variation in mortality and age-related disease explained by the exposome are therefore conservative estimates and probably underestimate the full influence of the exposome. Our proteomic age clock model only made use of plasma expression of roughly 3,000 proteins currently available in the UKB. Future clocks built from larger sets of proteins may provide greater coverage of age-related biological changes, possibly capturing biology relevant for other exposures or diseases.

A further limitation of our approach is that we only systematically tested for linear associations. Future research modeling non-linear associations of exposures may provide greater precision in describing relationships between exposures and health outcomes. We also did not test for gene–environment interactions, as although genes and environment undeniably have a joint influence on age-related disease, these analyses are susceptible to false positive findings^34^. Last, PRS as proxies for the inherited genetic component of each disease are works in progress that somewhat underestimate the actual polygenic risk. PRS also ignore rare variation in single genes, such as *BRCA1/2* and the amyloid precursor protein for AD, owing to their low frequency.

Our study also sheds light on a specific roadblock for exposome research that we have not resolved: when trying to validate our findings externally in the Rotterdam Study, we were not able to replicate many findings from the UKB owing to lack of overlapping exposure assessments. Future studies assessing the exposome using blood-based biomarkers of exposures may solve this problem, but are beyond the scope of the present study.

Despite these limitations, we believe that our approach offers many advantages over traditional single exposure approaches in epidemiology. While the majority of exposures tested in our analysis have already been previously demonstrated to associate with risk of mortality, the novelty of our results come from (1) quantifying the contribution of all environmental variables available in the UKB together for explaining variation in mortality, aging and major age-related diseases, and (2) comparing the contribution of the exposome to that of age, sex and the genome using PRS. Setting our study in the UKB allowed us to (1) simultaneously study premature mortality and proteomic aging; (2) develop a pipeline that addressed the major challenges in exposome research (namely reverse causation, correlation confounding and multicollinearity), thus identifying exposures that are independently associated with mortality and aging; and (3) split the cohort into independent discovery, replication and validation stages across different populations with sufficient power. When compared with the only previously published ‘environment-wide’ analysis of mortality^35^ that focused on a narrower range of chemical and lifestyle exposures in a small sample (*n* = 6,008), our study identified approximately 17 times more factors associated with all-cause mortality and improved the final mortality variance explained (*R*^2^) by 31 times, from 2.1% in the previous study to 66%. This demonstrates the importance of using large datasets and testing as broad a range of exposome influences as possible.

Overall, our results indicate that environment-focused interventions are possibly the most strategic starting point for ameliorating premature mortality and most age-related morbidity, although future causal modeling will be needed to study specific exposures of interest. Our study underscores that large biobanks, such as the UKB, open the door for further targeted proteomic, metabolomic or other omics studies to understand the impact of the exposome and disentangle the interplay between genetic and environmental exposures in premature mortality and aging.

## Methods

### Study design and participants

The UKB is a prospective cohort study with extensive genetic and phenotype data available for 502,505 individuals resident in the UK^36^. The full UKB protocol^37^ is available online. All statistical analyses were carried out using R v.4.2.2. and PLINK v.2.0.

### Exposures

We considered all non-genetic variables available as of 24 July 2020 that were collected or derived (for example, air pollution and Townsend deprivation index) at baseline, had <80% missing, and were available for participants recruited across all assessment centers as potential XWAS exposures. After all exclusions, recoding and quality control (Supplementary Information and Supplementary Tables 9 and 10), 176 unique exposures remained that were available in the full cohort and were common to both women and men. All continuous exposure variables were centered and standardized before analysis, except for age at recruitment. All ordinal categorical variables were recoded to only test linear associations and other polynomial contrasts (for example, quadratic or cubic associations) were not assessed. All nominal categorical exposures were analyzed with the most common category set as the reference. All ‘mark all that apply’ questions were recoded as binary dummy variables. Detailed data dictionaries including all exposures used in imputation and XWAS steps are included in Supplementary Files 1 and 2.

### Outcomes

Detailed information about the linkage procedure^38^ with national registries for mortality and cause of death information is available online. Mortality data were accessed from the UKB data portal on 4 May 2022, with a censoring date of 30 September 2021 or 31 October 2021 for participants recruited in England/Scotland or Wales, respectively (11–15 years of follow-up).

Procedures for calculating proteomic aging in the UKB were described previously^19^. Aging biomarkers (Supplementary Table 6) were measured using baseline nonfasting blood serum samples as previously described^39^. Data on leukocyte telomere length were only available in a slightly smaller sample (*n* = 472,506) than other biomarkers and were not imputed. Biomarkers were previously adjusted for technical variation by the UKB, with sample processing^40^ and quality control^41^ procedures described on the UKB website.

Data used to define prevalent and incident cases for chronic diseases and common disease risk factors are outlined in Supplementary Table 8. Incident chronic disease diagnoses were ascertained using International Classification of Diseases (ICD) diagnosis codes and corresponding dates of diagnosis taken from linked hospital inpatient records and death register data. ICD-9 and ICD-10 data were accessed from the UKB data portal on 30 May 2022, with a censoring date of 30 September 2021, 31 July 2021 or 28 February 2018 for participants recruited in England, Scotland or Wales, respectively (8–15 years of follow-up). Breast, ovarian and prostate cancer analyses were carried out as sex-specific analyses in female (breast and ovarian) or male (prostate) participants.

### Missing data imputation

The average percentages of missing data across all final variables included in our UKB analysis datasets were 11% in women (range: 0–79%) and 10.9% in men (range: 0–77%). UKB participants recruited from England were randomly assigned to a discovery (*n* = 218,446) or replication set (*n* = 218,445) while maintaining the same proportion of mortality cases in each. We performed missing data imputation separately in the discovery, replication and Scottish/Welsh validation (*n* = 55,676) datasets using the R package missRanger^42^, which combines random forest imputation with predictive mean matching. We imputed five datasets, with a maximum of ten iterations for each imputation. We set the maximum number of trees for the random forest to 200, but left all other random forest hyperparameters at their default. The variables used as predictors in the imputation included all baseline, non-nested variables, the Nelson–Aalen estimate of cumulative mortality hazard and the all-cause mortality event indicator. All subsequent study analyses were run independently in each of the five imputed datasets, and results were pooled using Rubin’s rule^43^.

### XWAS

XWAS of all-cause mortality were initially carried out separately in women and men, and then a final XWAS was calculated in the pooled dataset with both women and men to increase power. Exposures in the final pooled XWAS were limited to those applicable to both women and men, omitting sex-specific reproductive factors (only tested in the sex-specific XWAS). In each XWAS, we serially assessed associations of each individual exposure with all-cause mortality using Cox proportional hazards models with age as the timescale stratified by 5-year birth cohorts and sex (in the pooled analysis only), and adjusted for assessment center, years of education (7 years, 10 years, 13 years, 15 years, 19 years and 20 years) and ethnicity (white, Asian, Black, mixed or other). For each model, the baseline hazards were calculated separately in each of these strata, and resulting effect estimates are those that fit best across all strata. Since it has been shown that UKB participants are likely to misreport alcohol consumption as a function of higher disease burden^44^, self-reported overall health status was added as an additional XWAS covariate for the self-reported alcohol intake exposure only. *P* values in the discovery and replication analyses were corrected using the FDR (Benjamini–Hochberg method^45^) with a significance threshold of FDR *P* < 0.05. After completing the mortality XWAS, discovery and replication sets were recombined into the full English sample (*n* = 436,891) to complete further sensitivity analyses.

### Prevalent disease sensitivity analysis

We conducted a sensitivity analysis in the full sample of participants recruited in England (*n* = 436,891) where we individually tested every exposure replicated in the pooled mortality XWAS again in relation to mortality using the same XWAS formula and covariates, but now adding an interaction term between each exposure and an indicator of baseline disease or poor health (see definition below). We flagged and removed from further analysis any exposure that no longer had a significant direct effect in this model (*P* < 0.05) but its interaction with the baseline poor health indicator was significant (*P* < 0.05).

The baseline disease/poor health indicator was created for all participants, in which participants were coded as having disease or poor health at baseline if they (1) had a linked hospital inpatient ICD diagnosis for any of the chronic illnesses or common disease risk factors studied in our analysis (including hypertension, dyslipidemia and obesity) with a diagnosis date before or on their date of recruitment to the UKB; (2) were assigned a diagnosis code for any of the chronic diseases or common disease risk factors studied in our analysis during the baseline clinical interview (field IDs 20001 and 20002 in Supplementary Table 8); (3) self-reported a physician diagnosis of heart attack (field ID 6150), angina (field ID 6150), stroke (field ID 6150), high blood pressure (field ID 6150), bronchitis/emphysema (field ID 6152), diabetes (field ID 2443) or cancer (field ID 2453); (4) self-reported ≥1 cancer diagnoses (field ID 134); (5) self-reported taking insulin medication (field IDs 6153 and 6177), cholesterol lowering medication (field IDs 6153 and 6177) or blood pressure medication (field IDs 6153 and 6177); or (6) self-reported their overall health status as ‘poor’ (field ID 2178).

### PheWAS of replicated exposures

For all exposures replicated in the XWAS and not removed during the above-described disease sensitivity analyses, a PheWAS was conducted. In each PheWAS, the exposure was used as the outcome variable (hereafter referred to as exposure outcomes) and was tested against the full set of baseline phenotypes available in the UKB (Supplementary File 62 provides the full list of phenotypes tested). Each PheWAS was conducted as a linear or logistic regression, depending on whether the exposure outcome was continuous or categorical, with covariates for age at recruitment and sex. All ordinal exposure outcomes were tested as continuous variables. Nominal categorical exposure outcomes were recoded into dummy variables for each response category versus the reference. All continuous phenotype exposures were scaled and centered to the mean before running the PheWAS. Summary statistics from all PheWAS are available in Supplementary Files 63–178.

### Proteomic age clock analyses

We serially assessed associations between each exposure and proteomic age gap (the difference in years between plasma protein-predicted age and calendar age) using cross-sectional linear regression models with covariates for sex, age at recruitment, assessment center, years of education and ethnicity. In brief, we previously developed a proteomic age clock in a subset of UKB participants (*n* = 45,441) using a gradient boosting machine learning model that takes 204 proteins we identified and uses them to accurately predict chronological age (Pearson *r* = 0.94)^19^. In a validation study involving biobanks in China (*n* = 3,977) and Finland (*n* = 1,990), the proteomic age clock showed similar age prediction accuracy (Pearson *r* = 0.92 and *r* = 0.94, respectively) compared with its performance in the UKB. The proteomic age clock has been previously associated with the incidence of 18 major chronic diseases (including diseases of the heart, liver, kidney and lung, diabetes, neurodegeneration and cancer), as well as with multimorbidity and all-cause mortality risk.

### Correlation and cluster analyses

Correlation between all variables was calculated in the full sample of participants recruited in England using the R package polycor^46^ to create a heterogeneous correlation matrix for each imputed dataset. Correlation coefficients were first calculated within each imputed dataset, transformed to a normally distributed *z*-score via Fisher’s *z* transformation, pooled via Rubin’s rule and then retransformed back to the original *r*-scale coefficient after pooling. We used hierarchical clustering via Euclidean distance to identify the cluster structure of exposures replicated in the pooled XWAS and not susceptible to reverse causation bias (plus education and ethnicity). We used within-cluster sum of squares (WSS) analyses to identify candidates for the optimal number of clusters. We first computed the hierarchical clustering of exposures for different numbers of clusters (*k*) ranging from 1 to 25. For each *k*, we then calculated the WSS. We plotted the WSS as a function of the number of clusters *k*, and examined the plot visually to find the elbow in the plot (Supplementary Fig. 2). We determined that a seven cluster solution was the best approximation of the elbow in the WSS curve and represented the most appropriate conceptual groupings of exposures. When visually inspecting the dendrogram of hierarchical correlation, seven clusters separate the variables very well in terms of breaking variables into discrete groups with large distances/heights between clusters.

We further conducted multivariable modeling within each of these seven clusters using the following procedure: (1) all exposures in the cluster were run in a single multivariable mortality Cox model to check for multicollinearity using the variance inflation factor. Exposures with a generalized variance inflation factor^(1/(2×d.f.))^ >1.6 were flagged for collinearity and removed. (2) After any collinear variables are removed, all remaining exposures in the cluster were tested together in a single multivariable mortality Cox model using age as the timescale, stratified by 5-year birth cohorts and sex, and adjusted for UKB assessment center, household income (less than £18,000, £18,000–£30,999, £31,000–£51,999, £52,000–£100,000, greater than £100,000), education and ethnicity (if those variables were not already in the cluster). Significance in all the cluster multivariable models was determined by a nominal *P* < 0.05.

### Aging mechanisms and incident chronic disease analyses

Aging biomarker variables (more details in Supplementary Tables 6 and 7) were log transformed and then were age-adjusted by regressing each onto age at recruitment separately in women and men. Across exposures replicated in the XWAS and passing all sensitivity tests, we serially assessed associations between each exposure and age-adjusted biomarker using cross-sectional linear regression models with covariates for sex, 5-year birth cohort, assessment center, years of education, ethnicity, number of medications, smoking status (current, previous or never) and IPAQ physical activity level (low, moderate or high). Insulin-like growth factor 1 (IGF-1), leukocyte telomere length and vitamin D models included additional covariates for standing height (in cm), leukocyte count (10^9^ cells per liter) and month of biomarker assessment (to control for seasonality of sun exposure), respectively.

For chronic disease analyses, we serially assessed associations between each exposure (replicated in the mortality XWAS and surviving the disease sensitivity and cluster modeling stages) and incident disease using a Cox proportional hazards model, with all XWAS covariates plus household income, smoking status and IPAQ physical activity group. Sex-specific reproductive exposures (for example, menopause) replicated in the female- and male-only XWAS analyses were also tested as exposures in analyses of sex-specific chronic disease outcomes (breast, ovarian and prostate cancer).

For common disease risk factors (obesity, hypertension and dyslipidemia), we serially assessed each exposure and risk factor pair using cross-sectional logistic regression models adjusted for age, sex, assessment center, household income, years of education, ethnicity, smoking status and IPAQ physical activity level.

Across all biomarker, chronic disease, and common disease risk factor analyses, *P* values were corrected separately for each outcome using FDR.

### Calculating PRS

Where possible, we used multiancestry PRS that were previously made available by the UKB (Supplementary Table 11). Methods for deriving these PRS are described elsewhere^47^. For cancer outcomes where no PRS were provided by the UKB, we identified recent PRS from the Polygenic Score (PGS) catalog^48^, selecting scores derived in predominantly European populations that did not overlap with the UKB cohort (as no multiancestry scores were available). We calculated these PRS as weighted sums, ∑(no. risk alleles × effect size) in the UKB v3 imputed genotype data. PGS catalog entries used to calculate PRS were as follows: leukemia (PGS000077) by Graff et al.^49^, lung cancer (PGS000078) by Graff et al.^49^, pancreatic cancer (PGS000083) by Graff et al.^49^, esophageal cancer (PGS002298) by Choi et al.^50^, COPD score (PGS001788) by Wang et al.^51^, chronic kidney disease (PGS000859) by Mansour Aly et al.^52^, nonalcoholic fatty liver disease (PGS002282) by Schnurr et al.^53^, liver cirrhosis (PGS000726) by Emdin et al.^54^ and knee osteoarthritis (PGS002729) by Sedaghati-Khayat et al.^55^. All variants in these scores met our quality control criteria of imputation information >0.4 and minor allele frequency >0.005 in the UKB data. Although these new PRS were mostly developed in European populations, we calculated the PRS for our full multiancestry sample and accepted the limitation that the PRS may be slightly misspecified in non-European participants. All PRS were calculated using PLINK version 2.0.

All PRS were coded as quintiles for use in our multivariable models. In all multivariable models including PRS variables, we also added an additional covariate for genotype array (BiLEVE versus Axiom; field ID 22000) as well as the first 20 genetic principal components published by the UKB (field ID 22009).

### Exposome and polygenic risk multivariable models

For each outcome, five multivariable models were calculated. The first only includes age (scaled) and sex in the model (model 1). Model 2 includes age, sex and the PRS for the outcome, if available (see below for more detail). Model 3 includes age, sex and all exposures associated with the outcome (exposome). Model 4 includes age, sex, exposome and PRS. If a PRS was not available for a particular outcome, then models 2 and 4 were not calculated for that outcome. Each model was validated in the independent Scottish/Welsh dataset (*n* = 55,676) by obtaining the linear predicted values from the models in the English dataset and measuring the C-index and *R*^2^ for these values in relation to the outcome rates in the Scottish/Welsh population. For sex-specific outcomes (breast, ovarian and prostate cancers), we also included in the exposome all sex-specific exposures that were replicated in the female- and male-only mortality XWAS.

The Cox proportional hazards models used for these multivariable models differed slightly from those used in previous analyses, instead using time in study as the timescale, using recruitment age and sex as fixed covariates, and removing the 5-year birth cohort covariate from the model given its collinearity with age. In all multivariable Cox models, the proportional hazards assumption was tested by examining the Schoenfeld residuals, and an interaction with time was added to any variable with nonproportional hazards. Survival time splitting to use for time interactions in these models was performed using the timeSplitter function from the Greg R package^56^, using 2 years as the interval for time splitting. Any categorical exposure with less than ten outcome cases for one of the response levels was completely excluded from all exposome models for that specific outcome. The only exception was the variable on type of accommodation lived in, where instead we recoded all responses of ‘mobile or temporary structure (that is, caravan)’ to NA and removed that as a response level from the variable (since only a few hundred people endorsed this response level in the subset of participants in the multivariable models).

The *R*^2^ values for each model were calculated using the CoxR2 package^57^ as a measure of explained randomness based on the partial likelihood ratio statistic under the Cox proportional hazard model^58^. Following previous guidance^59^, *R*^2^ values were first calculated separately within each imputed dataset, converted to *r*-scale coefficients by taking the square root and then converted to the *z*-scale using Fisher’s *z* transformation. The *z*-transformed *R*^2^ values were then averaged across all five imputed datasets. These averaged values were then retransformed back to the *r*-scale using inverse *z* transformation and then squared to return a pooled *R*^2^ value. C-index values were also pooled using the same method. Relative importance for each variable and category of variables within the multivariable models was calculated using Wald *Χ*^2^ statistics via analysis of variance (ANOVA) using the rms package in R (ref. ^60^), where the relative importance of each is the proportion of the variable/group *Χ*^2^ relative to the total model *Χ*^2^.

### Ethics approval

UKB data use (project application no. 61054) was approved by the UKB according to their established access procedures. The UKB has approval from the North West Multi-center research ethics committee as a Research Tissue Bank, and as such researchers using UKB data do not require separate ethical clearance and can operate under the Research Tissue Bank approval.

### Reporting summary

Further information on research design is available in the Nature Portfolio Reporting Summary linked to this article.

## Online content

Any methods, additional references, Nature Portfolio reporting summaries, source data, extended data, supplementary information, acknowledgments, peer review information; details of author contributions and competing interests; and statements of data and code availability are available at 10.1038/s41591-024-03483-9.

## Supplementary information

Supplementary Information Supplementary Methods, Figs. 1 and 2, Tables 1–16, file legends and references.

Reporting Summary

Supplementary Files 1–178 Supplementary Files SF1–SF178.

## Figures and Tables

**Extended Data Table 1:**
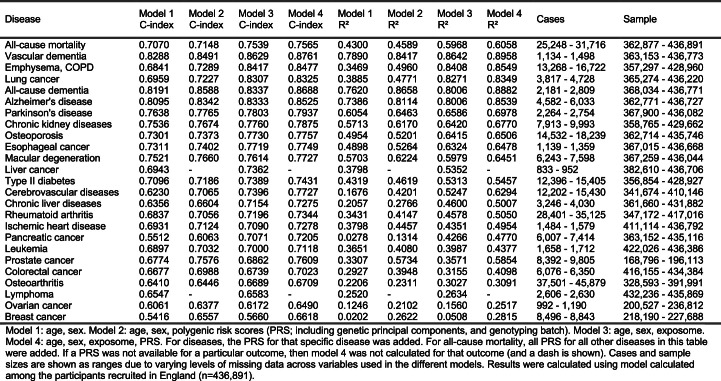
Explained variation and C-index across multivariable models in UK Biobank participants recruited in England

**Extended Data Table 2:**
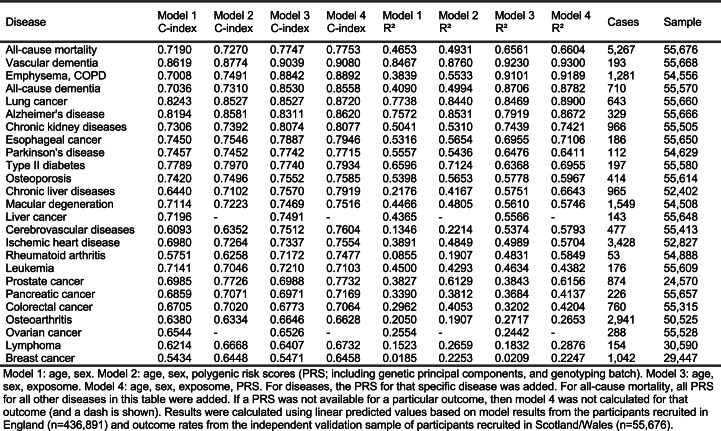
Explained variation and C-index across multivariable models in UK Biobank participants recruited in Scotland/Wales

## Data Availability

UKB data are available through a procedure described at https://www.ukbiobank.ac.uk/enable-your-research. Summary statistics from all analysis stages are included in Supplementary Files 3–178. All polygenic risk score summary statistics taken from the PGS catalog are publicly available at https://www.pgscatalog.org/.

## Code availability

R and PLINK code needed to reproduce all analyses, figures and tables are publicly available via GitHub at https://github.com/miargentieri/exposome-aging-ukb.
