# Supplementary material for: Integrating the environmental and genetic architectures of aging and mortality
Source: Nat Med. 2025 Feb 19;31(3):1016–25. doi: 10.1038/s41591-024-03483-9 (PMC11922759; doi:10.1038/s41591-024-03483-9)
Supplement: Supplementary file 1 — Supplementary Methods, Figs. 1 and 2, Tables 1–16, file legends and references. [file 41591_2024_3483_MOESM1_ESM.pdf]

nature medicine

Supplementary information https://doi.org/10.1038/s41591-024-03483-9

Integrating the environmental and genetic architectures of aging and mortality

In the format provided by the authors and unedited

Integrating the environmental and genetic architectures of mortality and aging

Argentieri et al. 2024

SUPPLEMENTARY INFORMATION

Supplementary methods ... 2
1.1 Variable coding ... 2
1.2 Derived variables ... 5
1.3 Variable quality control ... 10
1.4 Sensitivity analyses ... 11
Supplementary figures ... 13
Supplementary tables ... 16
Supplementary File legends ... 32
References ... 34

Supplementary methods

1.1 Variable coding

The following variable recoding was carried out before multiple imputation. All variable responses of “Prefer not to answer”, “Do not know”, and “Not applicable” were recoded to NA. Following multiple imputation, any variable values where the participant responded “Prefer not to answer” in the original dataset were recoded to NA. All nominal categorical variables were coded as unordered factors, with the reference level set as the most frequent response reported in the UK Biobank dataset. Ordered categorical variables were coded with the reference level set as the lowest response (e.g., “Never”, “Rarely”, or “None”). Dichotomous categorical variables were coded with the reference level set as “No.” All variables with “mark all that apply” response categories were converted into multiple dummy variables, with each unique response option used to create a yes/no dichotomous variable.

For all numeric diet intake variables, participants who responded “less than one” were recoded as 0.5. For variables on number of hours spent watching TV, on the computer, and driving, participants who responded “less than one” were recoded as 0.5.

To harmonize responses to the home area population density variable (field ID 20118) across different countries, this variable was recoded with a simpler classification of urban or rural home area population density. Participants who were classified in the original variable as "England/Wales - Urban – sparse," "England/Wales - Urban - less sparse," "Scotland - Large Urban Area," or "Scotland - Other Urban Area" were recoded to “Urban.” All other responses were recoded to “Rural,” with the exception of “Postcode not linkable.” Those with values of “Postcode not linkable” were set as NA after imputation.

All nested variables were excluded from analysis, except for several variables where we could recode to account for nesting. Two variables were recoded in this way before imputation and then imputed normally. For smoking pack years (field ID 20161) and smoking pack years as a proportion of life span exposed to smoking (field ID 20162), missing values were recoded as 0 if the respondent was coded as “No” in response to the derived ever smoked variable available in the UK Biobank (field ID 20160), but otherwise left as NA if respondents had ever smoked.

We also recoded several nested variables after multiple imputation. For bread type, cereal type, and coffee type variables, all participants with a response of 0 for the bread, cereal, and coffee intake questions were coded to “Never eat bread”, “Never eat cereal,” and “Never drink coffee,” respectively. After imputation, we also recoded the number of people living in your household variable to NA if a respondent reported living in a care home or sheltered accommodation, since that question was not asked to these participants and there were very few of them. We then recoded dummy variables created from field ID 6141 (how are you related to people in your household). Specifically, if the participant reported being the only person living in their household, then each dummy variable was set to “No.” Additionally, if the participant reported living in a care home or sheltered accommodation, then each dummy variable was set to NA. For example, for the yes/no dummy variable indicating whether the participant lives at home with parents, the response was recoded to “No” if that same participant reported living as the only person in their household and was set to NA if that same participant reported living in a care home or sheltered accommodation.

Non-exposome response level recoding. For two nominal categorical variables, we removed a specific level as we deemed that it was capturing disability and not the exposome. The first was Type of accommodation lived in (field ID 670), where we recoded anyone reported living in “sheltered accommodation” to NA and removed that response level from the variable.

The second was Major dietary changes in the last 5 years (field ID 1538), where we recoded anyone reported “Yes, because of illness” to NA and removed that response level from the variable.

Diet variable recoding. Following previous research on diet in the UK Biobank [1], we recoded a number of diet variables. For processed meat (field ID 1349), poultry (field ID 1359), oily fish (field ID 1329), and non-oily fish (field ID 1339), we combined the top three frequencies to get four categories: never, < 1.0 time per week, 1.0 time per week, and ≥ 2.0 times per week. For cheese intake (field ID 1408), we combined the bottom two frequencies and the top two frequencies to get four categories: < 1.0 time per week, 1.0 time per week, 2.0-4.9 times per week, and ≥ 5.0 times per week. For daily tea intake (field ID 1488), we grouped participants into the following categories: < 2.0 cups per day, 2.0-3.9 cups per day, 4.0-5.9 cups per day, and ≥ 6.0 cups per day. For daily coffee intake (field ID 1498), we grouped participants into the following categories: 0 cups/day, 0.5-1.9 cups per day, 2.0-2.9 cups per day, and ≥ 3.0 cups per day. Cereal (field ID 1458), bread (field ID 1448), and water (field ID 1528) intake were categorized into quartiles based on participants’ responses. Cereal was coded as < 2 bowls/week, 2-4.9 bowls/week, 5-6.9 bowls/week, ≥ 7 bowls/week. Bread was coded as <8 slices/week, 8-13.9 slices/week, 14-19.9 slices/week, ≥ 20 slices/week. Water was coded as <1 glass/day, 1-1.9 glass/day, 2-2.9 glass/day, ≥ 3 glass/day.

Finally, in line with previous large-scale research on alcohol consumption [2], we restricted analyses of alcohol intake frequency (field ID 1558) to current drinkers only. Previous research has shown that including previous drinkers and never drinkers together in measures of current alcohol intake frequency leads to substantial confounding in mortality analyses due to the fact that many previous drinkers are those that stopped drinking due to some health issue or disability [3,4]. For alcohol intake, all participants who responded as “Never” or “Previous” drinkers

to the alcohol status variable (field ID 20117) were coded as NA. In addition, we coded participants who responded as drinking on “Special occasions only” as NA. The final variable was coded as a nominal variable with responses for “One to three times a month”, “Once or twice a week”, “Three or four times a week”, and “Daily or almost daily”, with “One to three times a month” set as the reference.

1.2 Derived variables

Several derived variables were calculated in our UK Biobank dataset. All derived variables were calculated after imputation, and the original variables used to create each of the derived variables were then excluded from the XWAS analysis (with the exception of diet variables used to construct the partial fiber score).

Ethnicity. Responses to the baseline UK Biobank self-reported ethnicity question (field ID 21000) were condensed into Black, Asian, white, mixed, and other response categories. This condensation largely follows previous research in the UK Biobank [5], although further collapses all reported Asian ethnic backgrounds into a single response category. Response categories were re-coded as follows: Black = "Black or Black British", "Caribbean”, "African", "Any other Black background"; White = "White", "British", "Irish”, "Any other white background"; Mixed = "Mixed”, "White and Black Caribbean", "White and Black African”, "White and Asian", "Any other mixed background"; Asian="Asian or Asian British", "Chinese", "Indian", "Pakistani, "Bangladeshi", "Any other Asian background"; Other = "Other ethnic group”.

Sleep. We created a categorical variable for hours of sleep using the UK Biobank hours of sleep continuous measure (field ID 1160). In line with recommendations from the American

Academy of Sleep Medicine and Sleep Research Society [6], category levels used for hours of sleep were <7 hours, 7-9 hours, and >9 hours, with the reference set as 7-9 hours.

Education. Following previous research on education in the UK Biobank [7-9], an education years variable was created by converting the responses from the education qualifications variable (field ID 6138) into the equivalent years of education. Response categories were mapped onto years of education using the International Standard Classification of Education (ISCED) scale as follows: 7 years = "none of the above (no qualifications)"; 10 years = "CSEs or equivalent" or "O levels/GCSEs or equivalent"; 13 years = "A levels/AS levels or equivalent"; 15 years = "other professional qualification"; 19 years = "NVQ or HNC or equivalent"; 20 years = "college or university degree." Where participants marked multiple educational qualifications, the qualification with the highest corresponding years of education was used for that participant.

Standardized lung function. Standardized FEV1 and FVC variables were created by dividing the FEV1 (field ID 20150) and FVC (field ID 20151) best measure variables by standing height squared (field ID 50). This was done to ensure that values for these variables were not strongly determined by body size [10,11].

Normalized hand grip strength. Hand grip strength variables (field IDs 46-47) were normalized to body mass by dividing by weight [12,13].

Bread and cereal fiber scores. Following a method previously reported in the UK Biobank [14], we created bread and cereal fiber scores using baseline self-report data on intake of bread type and bread intake, and breakfast cereal type and breakfast cereal intake. Bread and cereal intake were measured as a numeric response corresponding to portions consumed per week. Bread and cereal intake were divided by 7 to get an estimate of daily intake, and this daily intake was multiplied by the estimated fiber content for the specific type of bread and cereal that

each participant reported to mainly eat. Participants who indicated “less than one” in response to the weekly bread and cereal intake questions were recoded as 0.5.

We converted our continuous fiber scores into quintiles and analyzed bread and cereal fiber as an ordinal variables. After creating the bread and cereal fiber scores, consumption of the individual food components of the score were not retained for further analyses.

Total red meat consumption. We created a new total red meat consumption variable by summing the frequencies for beef (field ID 1369), pork (field ID 1389), and lamb/mutton (field ID 1379), using the following coding: ‘Never’ = 0, ‘Less than once a week’ = 0.5, ‘Once a week’ = 1, ‘2-4 times a week’ = 3, ‘5-6 times a week’ = 5.5, ‘Once or more daily’ = 7. These values were then summed across the three variables and participants were coded into 4 categories of total red meat consumption: <1 time per week, 1.0-1.9 times per week, 2.0-2.9 times per week, and ≥3.0 times per week.

Total fruit consumption. Participants were asked to enter the number of pieces of fresh fruit (field ID 1309) and dried fruit (field ID 1319) they eat per day. One piece of fresh fruit, and two ‘pieces’ of dried fruit were counted as a serving. We then summed the number of total servings consumed per day and grouped participants into the following categories: < 2.0 servings per day, 2.0-2.9 servings per day, 3.0-3.9 servings per day, and ≥ 4.0 servings per day.

Total vegetable consumption. Participants were asked to enter the number of heaped tablespoons of cooked vegetables (field ID 1289) and salad/raw vegetables (field ID 1299) they eat per day. Two heaped tablespoons of either type of vegetables were counted as a serving. We then summed the number of total servings consumed per day and grouped participants into the following categories: < 2.0 servings per day, 2.0-2.9 servings per day, 3.0-3.9 servings per day, and ≥ 4.0 servings per day.

Total dairy milk intake. We derived an estimate of total dairy milk intake using the questions on type of milk (field ID 1418), bowls of breakfast cereal (field ID 1458), cups of tea (field ID 1488), and cups of coffee (field ID 1498). For participants who selected one of 'Full cream', 'Semi-skimmed' or 'Skimmed' milk intake, we calculated their total daily dairy milk consumption by summing 100 mL of milk for each bowl of breakfast cereal, 35 mL of milk for each cup of tea, and 25 mL of milk for each cup of coffee. Participants were then divided into three categories: those that consumed < 150 mL of milk, 150-299 mL of milk, and ≥ 300 mL of milk daily. Participants who answered 'Never/rarely have milk' to the question on type of milk consumed were assigned to the first category. This derivation of totally daily dairy milk intake was previously shown in the UK Biobank to discriminate well between those who had low and high dairy milk intakes according to the more detailed 24-hour dietary assessment variables [1].

Leisure time physical activity. As has been done previously [15], we used responses from a mark all that apply question (field ID 6164) asking participants about types of physical activity they have undertaken in the past 4 weeks to create a summary leisure time physical activity (LTPA) score. For each type of activity, if the participant reported undertaking that activity in the past 4 weeks then they were asked how many times in the past 4 weeks and the how long they spent on the activity each time. Those responses were used to determine the number of days/week for each activity and the number of minutes for each activity. These values were multiplied and divided by 7 to get the mins/day for each activity, which was multiplied by the metabolic equivalent of task (MET) for each activity. We then summed these values across all variables to get a total LTPA MET value for each participant. Participants were categorized into three groups of LTPA using the IPAQ scoring system (https://www.physio-pedia.com/images/c/c7/Quidelines_for_interpreting_the_IPAQ.pdf): (1) high activity: 3,000 MET-

mins per week or greater; (2) moderate: 600-3,000 MET-mins per week; and (3) low: less than 600 MET-mins per week.

Occupational physical activity. As has been done previously [15], we used responses on whether participants' work involves heavy manual or physical work (field ID 816) or involves mainly walking or standing (field ID 806) to create a summary occupational physical activity (OPA) score. For both variables, participants reported how often they performed each type of work by choosing from the following categories: "Never/rarely", "Sometimes," "Usually," or "Always." To estimate minutes spent per week in each of these types of work, we first multiplied the hours of employment per week (field ID 767) by 60 to get the total minutes per week that each participant spent at work. Values of mins of employment per week were set to 0 for all participants who did not indicate that they were in paid employment or self-employed (field ID 6142). We then calculated the number of minutes spent specifically in each type of heavy manual and walking/standing work by adjusting the total minutes of work per week according to participants responses to how frequently they engage in heavy manual or walking/standing work. Specifically, for both variables we multiplied the minutes spent working per week by: 0 if the participant replied "Never/rarely"; 1/3 if the participant replied "Sometimes"; 2/3 if the participant replied "Usually"; and 1 if the participant replied "Always." In this way, we calculated the approximate minutes per week that each participant spent in both heavy manual and walking/standing work. The mins per week for both types of work were then multiplied by the metabolic equivalent of task (MET) for each activity and these values were summed across both variables to get a total OPA MET value for each participant. We used the same MET thresholds per week as the LTPA variable to categorize participants into low, moderate, and high OPA.

Total sedentary time. Total sedentary time was measured according to a previously reported method in the UK Biobank [16], using measures on self-reported hours spent on a typical

day watching television (field ID 1070), using the computer (field ID 1080), and driving (field ID 1090). Values for each variable greater than 24 hours per day were excluded, and those reporting over 16 hours were re-coded to 16 hours. Tertiles were used to categorize sedentary time into low (0-4 hours), medium (5-6 hours), and high (>6 hours) levels of sedentary behavior, with 0-4 hours set as the reference and the variable was classed as an unordered factor.

1.3 Variable quality control

As a final quality control (QC) step conducted after imputation but before running the sex-specific XWAS, we systematically examined the crosstabs between each potential categorical exposure variable and the mortality binary indicator in both women and men. Any categorical variable with less than 10 mortality cases for a single response level was then flagged for further inspection. Three possible quality control actions were then undertaken:

(1) Binary categorical variables with less than 10 mortality cases for one of the response levels were completely excluded from the XWAS.

(2) We collapsed the “All of the time” and “Often” response levels for the narcolepsy variable, and we also collapsed the “Yes” and “I am completely deaf” response levels for the variable “Do you have any difficulty with your hearing?”

(3) For nominal categorical variables, any response levels with too few mortality cases (n=5) were set to NA and therefore not analyzed in the XWAS. The only variable that this affected was the “What type of accommodation do you live in?” variable, where the response level indicating that participants were living in a care home was set to NA and not analyzed in the XWAS because too few participants endorsed that response.

In order to improve the interpretability of our results, all responses of “none of the above” or “other” for nominal variables were set to NA and not analyzed. The response level of “None of the above” was also set to NA for the usual walking pace variable, and the resulting response levels of "Brisk pace", "Steady average pace", and "Slow pace" were recoded to be an ordinal variable with slow pace as the highest response level. Usual walking pace was only used as an exposure in phenome-wide association study analyses and not in the XWAS.

1.4 Sensitivity analyses

Prostate cancer. After conducting analyses between all validated exposures and incident prostate cancer, we observed that many significant associations were in the opposite direction as expected. Notably, we observed all smoking variables showed an association with decreased risk of incident prostate cancer. We attempted to interrogate this by stratifying the sample population by those who had ever received a PSA test at baseline (field ID 2365; n=137,598 for those with no PSA test, n=58,425 for those with a PSA test), as well as conducting a model in the full sample of men recruited in England (n=196,113) with PSA test as a fixed covariate. All Cox models included age as the timescale, were stratified by 5-year birth cohorts, and were adjusted for UK Biobank assessment center, household income, ethnicity, years of education, and IPAQ activity group. All polynomial contrasts were allowed for all ordinal variables.

Accelerometer data and self-reported physical activity. We also calculated mortality multivariable models using the accelerometer data available in the UK Biobank. Data collection for accelerometer data occurred from 2013-2015 in a subsample of 103,672 UK Biobank participants. Mortality survival times were re-calculated using the date from the start time of the accelerometer wear period (field ID 90010) as the start date of follow up for each participant.

We used the overall acceleration average variable (field ID 90012) as our measure of objective physical activity. Description of the development of this variable has been described previously [17]. Before analysis, we carried out several quality control steps to refine the overall acceleration average variable by excluding participants: (1) whose data could not be calibrated (field ID 90016); (2) who did not wear the device long enough to get a stable measure of their physical activity status (field ID 90015); (3) with >1% clipped values before or after calibration (field IDs 90183, 90185) out of the total data readings (field ID 90187); or (4) who had an unrealistically high overall acceleration average of 100 milli-gravity or greater. This process excluded 6,189 participants, leaving a total sample of n=85,520 for the accelerometer analyses.

Two models were tested: the first included all self-reported baseline physical activity measures (IPAQ, LTPA, OPA, sedentary time) in the full sample of UK Biobank participants recruited in England (n=436,891), the second only included overall acceleration average in the subsample of participants with accelerometer data (n=85,520). Both models were run as Cox proportional hazards models with mortality survival time as the timescale with additional covariates for age at time of activity measurement, sex, UK Biobank assessment center, years of education, household income, ethnicity, smoking status, and Townsend deprivation index. All polynomial contrasts were allowed for all ordinal variables. When putting all self-reported baseline physical activity measures in the same model there was no collinearity according to VIF.

Supplementary figures

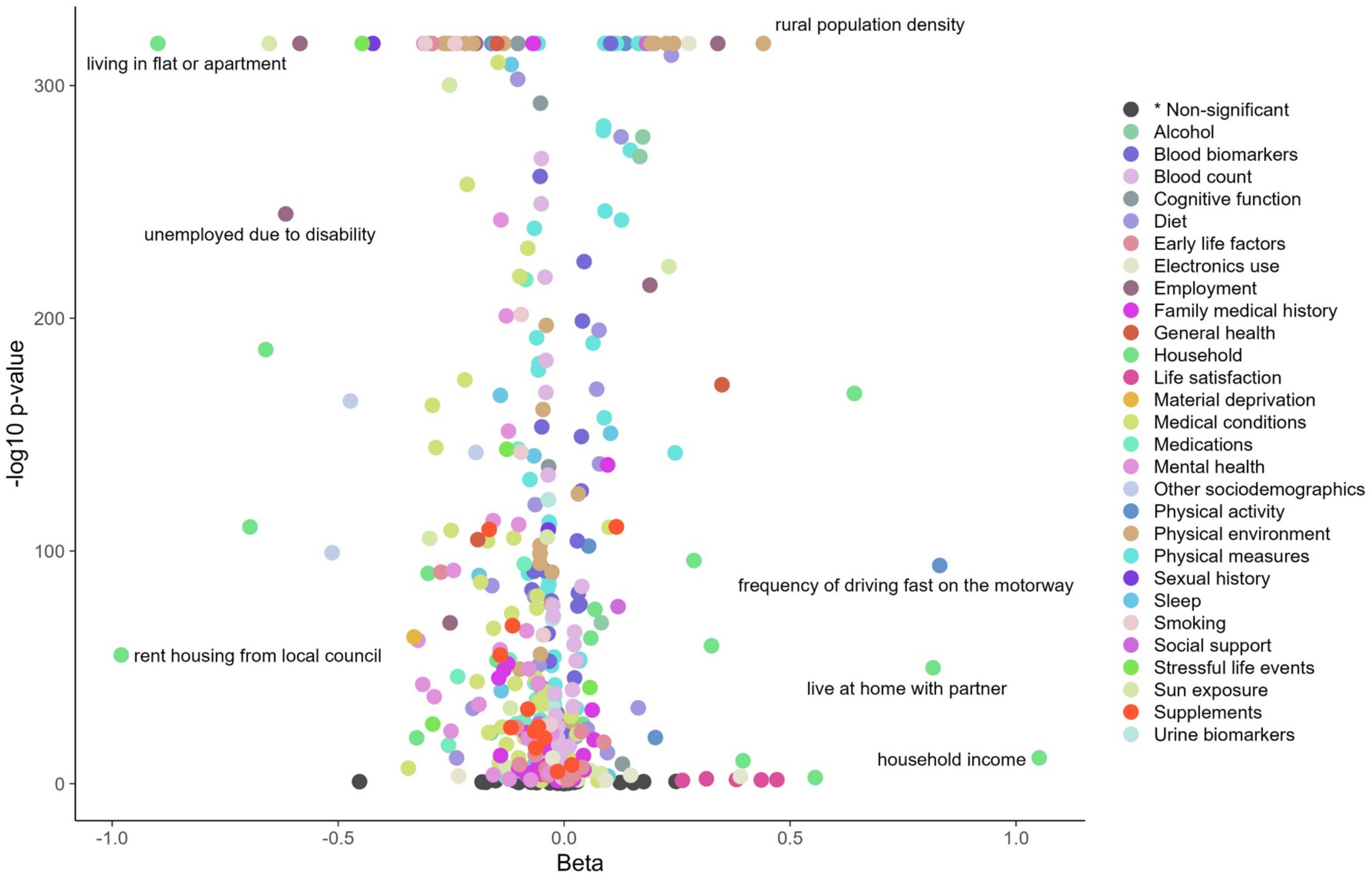

Supplementary Figure 1. Volcano plot of log-transformed p-values and fold change (calculated as $\log_2$ of the odds ratio) from a PheWAS of number of household vehicles in participants recruited in England (n=436,891). Each point represents the effect and p-value from a linear regression testing the association between a single exposure and number of household vehicles. P-values are corrected for multiple testing using the False Discovery Rate (FDR) threshold of 0.05. Exposures that were FDR significant are colored, whereas associations that were not significant are colored dark grey and grouped in the category "* Non-significant."

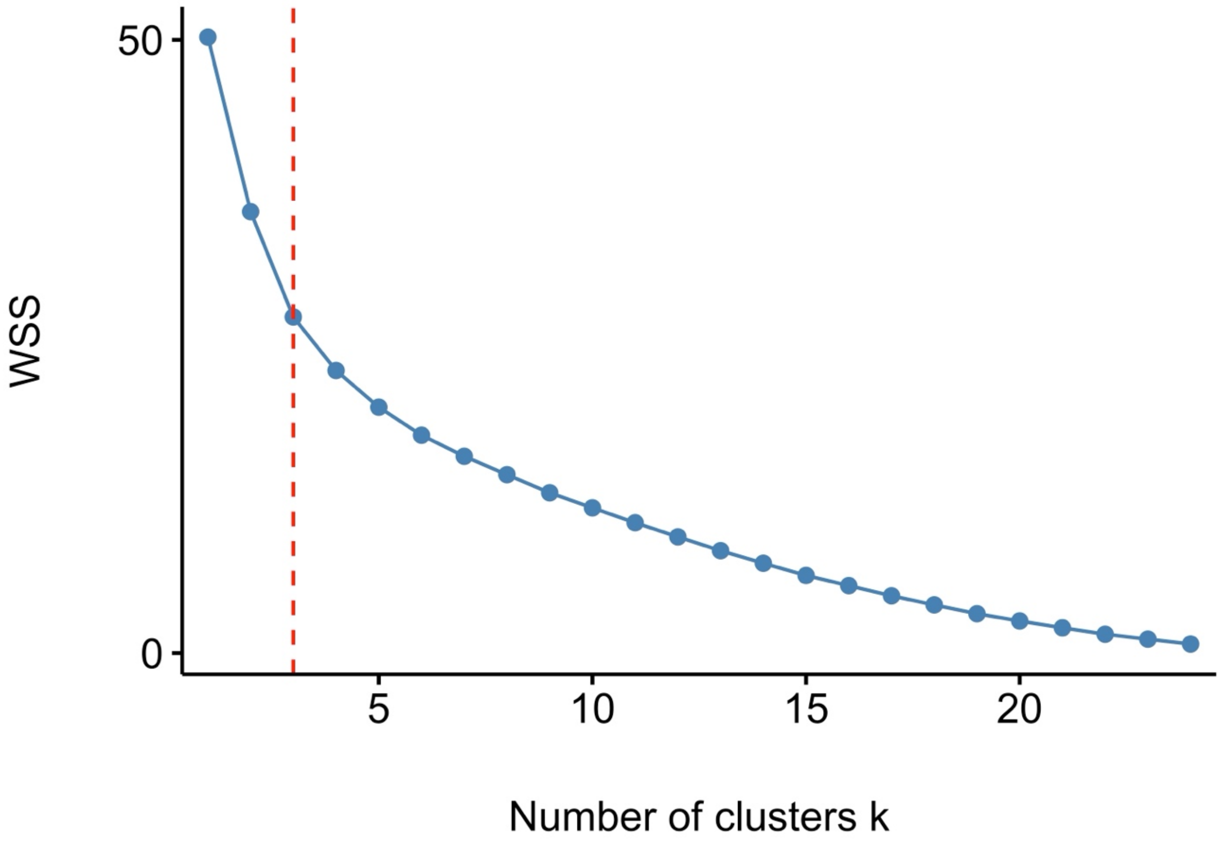

Supplementary Figure 2. Total within-cluster sum of squares (WSS; y-axis) according to the number of hierarchical clusters (y-axis) for exposures replicated in the pooled mortality XWAS. The red dotted line at 3 shows the number of clusters selected by AIC, however we selected 7 as a more appropriate number of clusters.

Supplementary tables

Supplementary Table 1. Baseline descriptive statistics - UK Biobank participants with no prevalent disease

	Female (N=163,415)	Male (N=137,905)	Total (N=301,320)
**Age**			
Mean (SD)	55 (8.0)	55 (8.2)	55 (8.1)
**Household income**			
Less than 18,000	29,254 (17.9%)	20,104 (14.6%)	49,358 (16.4%)
18,000 to 30,999	39,009 (23.9%)	30,209 (21.9%)	69,218 (23.0%)
31,000 to 51,999	39,122 (23.9%)	36,101 (26.2%)	75,223 (25.0%)
52,000 to 100,000	30,113 (18.4%)	31,891 (23.1%)	62,004 (20.6%)
Greater than 100,000	7,984 (4.9%)	9,057 (6.6%)	17,041 (5.7%)
**Education years**			
7 years	21,857 (13.4%)	18,072 (13.1%)	39,929 (13.3%)
10 years	32,872 (20.1%)	19,198 (13.9%)	52,070 (17.3%)
13 years	10,116 (6.2%)	7,321 (5.3%)	17,437 (5.8%)
15 years	21,435 (13.1%)	13,934 (10.1%)	35,369 (11.7%)
19 years	21,210 (13.0%)	27,055 (19.6%)	48,265 (16.0%)
20 years	54,466 (33.3%)	51,046 (37.0%)	105,512 (35.0%)
**Ethnicity**			
White	153,607 (94.0%)	129,698 (94.0%)	283,305 (94.0%)
Asian	3,481 (2.1%)	3,416 (2.5%)	6,897 (2.3%)
Black	3,095 (1.9%)	2,343 (1.7%)	5,438 (1.8%)
Mixed	1,185 (0.7%)	696 (0.5%)	1,881 (0.6%)
Other	1,647 (1.0%)	1,245 (0.9%)	2,892 (1.0%)
**BMI**			
Mean (SD)	26 (4.8)	27 (3.9)	27 (4.4)
**Smoking status**			
Never	100,764 (61.7%)	72,787 (52.8%)	173,551 (57.6%)
Previous	48,831 (29.9%)	48,644 (35.3%)	97,475 (32.3%)
Current	13,300 (8.1%)	16,046 (11.6%)	29,346 (9.7%)
**Home area population density**			
Urban	139,489 (85.4%)	117,969 (85.5%)	257,458 (85.4%)
Rural	23,926 (14.6%)	19,936 (14.5%)	43,862 (14.6%)
**Mortality**			
Alive	157,783 (96.6%)	129,820 (94.1%)	287,603 (95.4%)
Dead	5,632 (3.4%)	8,085 (5.9%)	13,717 (4.6%)

Baseline descriptive statistics for the subsample of UK Biobank participants with no disease at baseline (used for disease sensitivity analyses). Mortality rates are for the 11-15 year study follow up period. Means and percentages are calculated using the first imputed analysis dataset and are not pooled across imputed datasets. Standard deviation (SD) of the mean is shown for mean values.

Supplementary Table 2. Baseline descriptive statistics - UK Biobank participants recruited in Scotland/Wales

	Female (N=30,707)	Male (N=24,969)	Total (N=55,676)
**Age**			
Mean (SD)	56 (8.0)	57 (8.1)	56 (8.0)
**Household income**			
Less than 18,000	7,454 (24.3%)	4,843 (19.4%)	12,297 (22.1%)
18,000 to 30,999	7,060 (23.0%)	5,571 (22.3%)	12,631 (22.7%)
31,000 to 51,999	7,018 (22.9%)	6,370 (25.5%)	13,388 (24.0%)
52,000 to 100,000	5,037 (16.4%)	5,258 (21.1%)	10,295 (18.5%)
Greater than 100,000	1,127 (3.7%)	1,307 (5.2%)	2,434 (4.4%)
**Education years**			
7 years	5,571 (18.1%)	4,669 (18.7%)	10,240 (18.4%)
10 years	5,000 (16.3%)	2,828 (11.3%)	7,828 (14.1%)
13 years	1,893 (6.2%)	1,391 (5.6%)	3,284 (5.9%)
15 years	3,520 (11.5%)	2,453 (9.8%)	5,973 (10.7%)
19 years	3,466 (11.3%)	4,289 (17.2%)	7,755 (13.9%)
20 years	10,966 (35.7%)	9,104 (36.5%)	20,070 (36.0%)
**Ethnicity**			
White	30,101 (98.0%)	24,383 (97.7%)	54,484 (97.9%)
Asian	247 (0.8%)	266 (1.1%)	513 (0.9%)
Black	73 (0.2%)	72 (0.3%)	145 (0.3%)
Mixed	125 (0.4%)	86 (0.3%)	211 (0.4%)
Other	114 (0.4%)	104 (0.4%)	218 (0.4%)
**BMI**			
Mean (SD)	27 (5.3)	28 (4.3)	28 (4.9)
**Smoking status**			
Never	18,264 (59.5%)	12,575 (50.4%)	30,839 (55.4%)
Previous	9,221 (30.0%)	8,845 (35.4%)	18,066 (32.4%)
Current	3,128 (10.2%)	3,476 (13.9%)	6,604 (11.9%)
**Home area population density**			
Urban	27,481 (89.5%)	22,310 (89.4%)	49,791 (89.4%)
Rural	3,219 (10.5%)	2,648 (10.6%)	5,867 (10.5%)
**Mortality**			
Alive	28,518 (92.9%)	21,891 (87.7%)	50,409 (90.5%)
Dead	2,189 (7.1%)	3,078 (12.3%)	5,267 (9.5%)

Mortality rates are for the 11-15 year study follow up period. Descriptive statistics are calculated using the first imputed analysis dataset and are not pooled across imputed datasets. SD: standard deviation.

Supplementary Table 3. Mortality by cause of death - UK Biobank participants recruited in England

	Total deaths (N=31,716)
All diseases*	22,201 (70.0%)
Ischemic heart disease	5,320 (16.8%)
Lung cancer	2,845 (9.0%)
Cerebrovascular diseases	2,349 (7.4%)
Emphysema, COPD	2,266 (7.1%)
Colorectal cancer	1,624 (5.1%)
Breast cancer	1,404 (4.4%)
Type II diabetes	1,394 (4.4%)
All-cause dementia	1,281 (4.0%)
Pancreatic cancer	1,228 (3.9%)
Prostate cancer	1,216 (3.8%)
Chronic kidney diseases	1,160 (3.7%)
Esophageal cancer	816 (2.6%)
Lymphoma	764 (2.4%)
Parkinson's disease	722 (2.3%)
Chronic liver diseases	776 (2.4%)
Alzheimer's disease	661 (2.1%)
Leukemia	656 (2.1%)
Ovarian cancer	590 (1.9%)
Liver cancer	580 (1.8%)
Vascular dementia	362 (1.1%)
Rheumatoid arthritis	193 (0.6%)
Osteoporosis	63 (0.2%)
Osteoarthritis	36 (0.1%)
Macular degeneration	1 (0.0%)

Supplementary Table 4. Mortality by cause of death - UK Biobank participants recruited in Scotland/Wales

	Total deaths (N=5,267)
All diseases*	3,762 (71.4%)
Ischemic heart disease	936 (17.8%)
Lung cancer	558 (10.6%)
Cerebrovascular diseases	434 (8.2%)
Emphysema, COPD	400 (7.6%)
Type II diabetes	323 (6.1%)
Colorectal cancer	276 (5.2%)
All-cause dementia	251 (4.8%)
Breast cancer	216 (4.1%)
Pancreatic cancer	195 (3.7%)
Alzheimer's disease	181 (3.4%)
Chronic kidney diseases	178 (3.4%)
Prostate cancer	163 (3.1%)
Esophageal cancer	122 (2.3%)
Chronic liver diseases	129 (2.4%)
Lymphoma	112 (2.1%)
Parkinson's disease	106 (2.0%)
Liver cancer	103 (2.0%)
Ovarian cancer	97 (1.8%)
Vascular dementia	94 (1.8%)
Leukemia	91 (1.7%)
Rheumatoid arthritis	46 (0.9%)
Osteoporosis	12 (0.2%)
Osteoarthritis	3 (0.1%)
Macular degeneration	0 (0%)

Numbers and percentages represent the number of deaths for which each disease was listed as either the primary or contributory cause of death. Only diseases that were associated with at least one exposure are listed.

*All diseases encompasses only those diseases listed in this table and is an indicator of the number of participants with any of the diseases in this table listed as a primary or contributory cause of death

Supplementary Table 5. Chronic disease and clinical risk factor diagnosis rates - UK Biobank participants recruited in England

	Female (N=237,634)	Male (N=199,257)	Total (N=436,891)
**Colorectal cancer**			
No diagnosis	233,700 (98.3%)	194,334 (97.5%)	428,034 (98.0%)
Incident diagnosis	2,834 (1.2%)	3,516 (1.8%)	6,350 (1.5%)
Prevalent diagnosis	1,100 (0.5%)	1,407 (0.7%)	2,507 (0.6%)
**Lung cancer**			
No diagnosis	235,068 (98.9%)	196,424 (98.6%)	431,492 (98.8%)
Incident diagnosis	2,305 (1.0%)	2,423 (1.2%)	4,728 (1.1%)
Prevalent diagnosis	261 (0.1%)	410 (0.2%)	671 (0.2%)
**Esophageal cancer**			
No diagnosis	237,210 (99.8%)	198,099 (99.4%)	435,309 (99.6%)
Incident diagnosis	373 (0.2%)	986 (0.5%)	1,359 (0.3%)
Prevalent diagnosis	51 (0.0%)	172 (0.1%)	223 (0.1%)
**Liver cancer**			
No diagnosis	237,178 (99.8%)	198,576 (99.7%)	435,754 (99.7%)
Incident diagnosis	382 (0.2%)	570 (0.3%)	952 (0.2%)
Prevalent diagnosis	74 (0.0%)	111 (0.1%)	185 (0.0%)
**Pancreatic cancer**			
No diagnosis	236,855 (99.7%)	198,358 (99.5%)	435,213 (99.6%)
Incident diagnosis	736 (0.3%)	843 (0.4%)	1,579 (0.4%)
Prevalent diagnosis	43 (0.0%)	56 (0.0%)	99 (0.0%)
**Brain cancer**			
No diagnosis	237,133 (99.8%)	198,668 (99.7%)	435,801 (99.8%)
Incident diagnosis	379 (0.2%)	477 (0.2%)	856 (0.2%)
Prevalent diagnosis	122 (0.1%)	112 (0.1%)	234 (0.1%)
**Leukemia**			
No diagnosis	236,728 (99.6%)	197,946 (99.3%)	434,674 (99.5%)
Incident diagnosis	692 (0.3%)	1,020 (0.5%)	1,712 (0.4%)
Prevalent diagnosis	214 (0.1%)	291 (0.1%)	505 (0.1%)
**Lymphoma**			
No diagnosis	235,985 (99.3%)	197,254 (99.0%)	433,239 (99.2%)
Incident diagnosis	1,201 (0.5%)	1,429 (0.7%)	2,630 (0.6%)
Prevalent diagnosis	448 (0.2%)	574 (0.3%)	1,022 (0.2%)
**Breast cancer**			
No diagnosis	218,845 (92.1%)	199,105 (99.9%)	417,950 (95.7%)
Incident diagnosis	8,843 (3.7%)	90 (0.0%)	8,933 (2.0%)
Prevalent diagnosis	9,946 (4.2%)	62 (0.0%)	10,008 (2.3%)
**Ovarian cancer**			
No diagnosis	235,622 (99.2%)	199,254 (100.0%)	434,876 (99.5%)
Incident diagnosis	1,190 (0.5%)	3 (0.0%)	1,193 (0.3%)

Supplementary Table 5. Chronic disease and clinical risk factor diagnosis rates - UK Biobank participants recruited in England

	Female (N=237,634)	Male (N=199,257)	Total (N=436,891)
Prevalent diagnosis	822 (0.3%)	0 (0%)	822 (0.2%)
**Prostate cancer**			
No diagnosis	237,626 (100.0%)	186,308 (93.5%)	423,934 (97.0%)
Incident diagnosis	5 (0.0%)	9,805 (4.9%)	9,810 (2.2%)
Prevalent diagnosis	3 (0.0%)	3,144 (1.6%)	3,147 (0.7%)
**Type II diabetes**			
No diagnosis	220,185 (92.7%)	174,531 (87.6%)	394,716 (90.3%)
Incident diagnosis	6,838 (2.9%)	8,592 (4.3%)	15,430 (3.5%)
Prevalent diagnosis	10,611 (4.5%)	16,134 (8.1%)	26,745 (6.1%)
**Ischemic heart disease**			
No diagnosis	218,285 (91.9%)	163,606 (82.1%)	381,891 (87.4%)
Incident diagnosis	11,852 (5.0%)	19,369 (9.7%)	31,221 (7.1%)
Prevalent diagnosis	7,497 (3.2%)	16,282 (8.2%)	23,779 (5.4%)
**Cerebrovascular diseases**			
No diagnosis	227,488 (95.7%)	186,034 (93.4%)	413,522 (94.7%)
Incident diagnosis	6,765 (2.8%)	8,640 (4.3%)	15,405 (3.5%)
Prevalent diagnosis	3,381 (1.4%)	4,583 (2.3%)	7,964 (1.8%)
**Emphysema, COPD**			
No diagnosis	226,215 (95.2%)	186,023 (93.4%)	412,238 (94.4%)
Incident diagnosis	6,970 (2.9%)	8,485 (4.3%)	15,455 (3.5%)
Prevalent diagnosis	4,449 (1.9%)	4,749 (2.4%)	9,198 (2.1%)
**Chronic liver diseases**			
No diagnosis	233,239 (98.2%)	194,463 (97.6%)	427,702 (97.9%)
Incident diagnosis	3,621 (1.5%)	3,793 (1.9%)	7,414 (1.7%)
Prevalent diagnosis	774 (0.3%)	1,001 (0.5%)	1,775 (0.4%)
**Chronic kidney diseases**			
No diagnosis	228,299 (96.1%)	189,208 (95.0%)	417,507 (95.6%)
Incident diagnosis	8,858 (3.7%)	9,381 (4.7%)	18,239 (4.2%)
Prevalent diagnosis	477 (0.2%)	668 (0.3%)	1,145 (0.3%)
**All-cause dementia**			
No diagnosis	234,741 (98.8%)	195,953 (98.3%)	430,694 (98.6%)
Incident diagnosis	2,819 (1.2%)	3,214 (1.6%)	6,033 (1.4%)
Prevalent diagnosis	74 (0.0%)	90 (0.0%)	164 (0.0%)
**Vascular dementia**			
No diagnosis	236,967 (99.7%)	198,308 (99.5%)	435,275 (99.6%)
Incident diagnosis	614 (0.3%)	884 (0.4%)	1,498 (0.3%)
Prevalent diagnosis	53 (0.0%)	65 (0.0%)	118 (0.0%)
**Alzheimer's**			
No diagnosis	236,139 (99.4%)	197,823 (99.3%)	433,962 (99.3%)

Supplementary Table 5. Chronic disease and clinical risk factor diagnosis rates - UK Biobank participants recruited in England

	Female (N=237,634)	Male (N=199,257)	Total (N=436,891)
Incident diagnosis	1,441 (0.6%)	1,368 (0.7%)	2,809 (0.6%)
Prevalent diagnosis	54 (0.0%)	66 (0.0%)	120 (0.0%)
**Parkinson's**			
No diagnosis	236,302 (99.4%)	197,026 (98.9%)	433,328 (99.2%)
Incident diagnosis	1,035 (0.4%)	1,719 (0.9%)	2,754 (0.6%)
Prevalent diagnosis	297 (0.1%)	512 (0.3%)	809 (0.2%)
**Rheumatoid arthritis**			
No diagnosis	231,489 (97.4%)	196,363 (98.5%)	427,852 (97.9%)
Incident diagnosis	2,668 (1.1%)	1,362 (0.7%)	4,030 (0.9%)
Prevalent diagnosis	3,477 (1.5%)	1,532 (0.8%)	5,009 (1.1%)
**Macular degeneration**			
No diagnosis	232,478 (97.8%)	195,968 (98.3%)	428,446 (98.1%)
Incident diagnosis	4,625 (1.9%)	2,973 (1.5%)	7,598 (1.7%)
Prevalent diagnosis	531 (0.2%)	316 (0.2%)	847 (0.2%)
**Osteoporosis**			
No diagnosis	222,980 (93.8%)	196,689 (98.7%)	419,669 (96.1%)
Incident diagnosis	8,307 (3.5%)	1,686 (0.8%)	9,993 (2.3%)
Prevalent diagnosis	6,347 (2.7%)	882 (0.4%)	7,229 (1.7%)
**Osteoarthritis**			
No diagnosis	183,497 (77.2%)	162,615 (81.6%)	346,112 (79.2%)
Incident diagnosis	26,469 (11.1%)	19,410 (9.7%)	45,879 (10.5%)
Prevalent diagnosis	27,668 (11.6%)	17,232 (8.6%)	44,900 (10.3%)
**Hypertension**			
No diagnosis	45,992 (19.4%)	28,391 (14.2%)	74,383 (17.0%)
Prevalent diagnosis	191,642 (80.6%)	170,866 (85.8%)	362,508 (83.0%)
**Obesity**			
No diagnosis	181,877 (76.5%)	148,720 (74.6%)	330,597 (75.7%)
Prevalent diagnosis	55,757 (23.5%)	50,537 (25.4%)	106,294 (24.3%)
**Dyslipidemia**			
No diagnosis	108,118 (45.5%)	67,006 (33.6%)	175,124 (40.1%)
Prevalent diagnosis	129,516 (54.5%)	132,251 (66.4%)	261,767 (59.9%)

Prevalent chronic disease rates are calculated as those with a corresponding ICD diagnosis date before or on the date of recruitment into the UK Biobank cohort. Additionally, those who self-reported a physician diagnosis of cancer, diabetes, heart attack, stroke, or bronchitis/emphysema during the baseline verbal interview were also used to count prevalent cases for cancer, diabetes, heart disease, cerebrovascular disease, and chronic lower respiratory diseases, respectively. Incident disease rates are for the 11-14 year study follow up period and exclude those with prevalent disease at baseline. Descriptive statistics are calculated using the first imputed analysis dataset and are not pooled across imputed datasets.

Supplementary Table 6. Biomarker summary statistics by sex - UK Biobank participants recruited in England

	Female (N=237,634)	Male (N=199,257)	Total (N=436,891)
Alanine Aminotransferase (U/L)	20 (12)	27 (15)	23 (14)
Albumin (g/L)	45 (2.6)	46 (2.6)	45 (2.6)
Alkaline phosphatase (U/L)	85 (27)	82 (25)	84 (26)
Apolipoprotein A (g/L)	1.6 (0.27)	1.4 (0.23)	1.5 (0.28)
Apolipoprotein B (g/L)	1.0 (0.24)	1.0 (0.24)	1.0 (0.24)
Aspartate aminotransferase (U/L)	24 (9.4)	28 (12)	26 (11)
C-reactive protein (mg/L)	2.7 (4.4)	2.5 (4.4)	2.6 (4.4)
Cholesterol (mmol/L)	5.9 (1.1)	5.5 (1.1)	5.7 (1.1)
Creatinine (umol/L)	64 (14)	82 (19)	72 (19)
Cystatin C (mg/L)	0.88 (0.16)	0.94 (0.18)	0.91 (0.18)
Direct bilirubin (umol/L)	1.5 (0.64)	2.0 (0.93)	1.7 (0.82)
Gamma glutamyltransferase (U/L)	30 (34)	46 (50)	37 (42)
Glucose (mmol/L)	5.1 (1.1)	5.2 (1.4)	5.1 (1.2)
Glycated haemoglobin (HbA1c) (mmol/mol)	36 (5.9)	37 (7.6)	36 (6.7)
HDL cholesterol	1.6 (0.38)	1.3 (0.31)	1.5 (0.39)
Insulin-like growth factor 1 (IGF-1) (nmol/L)	21 (5.8)	22 (5.6)	21 (5.7)
LDL direct (mmol/L)	3.6 (0.87)	3.5 (0.86)	3.6 (0.87)
Leukocyte telomere length (T/S ratio)	0.84 (0.13)	0.82 (0.13)	0.83 (0.13)
Lipoprotein(a) (nmol/L)	45 (50)	44 (49)	45 (49)
Phosphate (mmol/L)	1.2 (0.15)	1.1 (0.16)	1.2 (0.16)
Total bilirubin (umol/L)	8.1 (3.7)	10 (4.9)	9.1 (4.4)
Triglycerides (mmol/L)	1.5 (0.85)	2.0 (1.2)	1.7 (1.0)
Urate (umol/L)	270 (66)	360 (72)	310 (81)
Urea (mmol/L)	5.2 (1.3)	5.6 (1.5)	5.4 (1.4)
Vitamin D (nmol/L)	49 (21)	49 (21)	49 (21)

Supplementary Table 7. Variables used for biomarker analyses

Biomarker	UK Biobank field ID
Alanine aminotransferase	30620
Albumin	30600
Alkaline phosphatase	30610
Apolipoprotein A	30630
Apolipoprotein B	30640
Aspartate aminotransferase	30650
High sensitivity C-reactive protein	30710
Cholesterol	30690
Creatinine	30700
Cystatin C	30720
Direct bilirubin	30660
Total bilirubin	30840
Gamma glutamyltransferase	30730
Glucose	30740
Glycated hemoglobin (hbA1c)	30750
HDL cholesterol	30760
Insulin-like growth factor 1 (IGF-1)	30770
LDL direct	30780
Lipoprotein A	30790
Phosphate	30810
Triglycerides	30870
Urate	30880
Urea	30670
Vitamin D	30890
Leukocyte telomere length (LTL)	22192

Supplementary Table 8. Variables used to calculate prevalence and incidence of chronic diseases and clinical risk factors

Chronic disease	Baseline measures (field ID)	Baseline verbal interview diagnosis codes	ICD-10 codes	ICD-9 codes
Colorectal cancer	-	1022, 1023	C18-C20	153, 154
Lung cancer	-	1001, 1027, 1028, 1080	C33, C34	162
Esophageal cancer	-	1017	C15	150
Liver cancer	-	1024	C22	155
Pancreatic cancer	-	1026	C25	157
Brain cancer	-	1032	C71	191
Leukemia	-	1048	C91-C95	204-208
Lymphoma	-	1047	C81-C86, C88	201-203
Breast cancer	-	1002	C50	174-175
Ovarian cancer	-	1039	C56	183
Prostate cancer	-	1044	C61	185
Type 2 diabetes	Taking insulin medication (6153, 6177)<br>Diabetes diagnosed by physician (2443)<br>Non-fasting blood hbA1c $\geq$ 48 mmol/mol (30750)<br>Non-fasting blood glucose $\geq$ 11.1 mmol/L (30740)	1223	E11	250
Ischemic heart disease	Heart attack diagnosis by physician (6150)<br>Angina diagnosis by physician (6150)	1074, 1075	I20-I25	410-414
Cerebrovascular diseases	Stroke diagnosis by physician (6150)	1081, 1086, 1491, 1583	I60-I69	430-438
Emphysema, COPD	Bronchitis/emphysema diagnosis by physician (6152)	1112, 1472	J43-J44	492
Chronic liver diseases	-	1157, 1158, 1604	K70, K73-K74, K75.8, K76.0	571
Chronic kidney diseases	-	1192, 1193, 1194	N18	585

All-cause dementia	-	1263	A81.0, F00-F03, F05.1, F10.6, G30-G31, I67.3	331.0, 290.4, 331.1, 290.2, 290.3, 291.2, 294.1, 331.2, 331.5
Vascular dementia	-	1263	F01, I67.3	290.4
Alzheimer's disease	-	1263	F00, G30	331.0
Parkinson's disease and parkinsonism	-	1262	G20-G22	332
Rheumatoid arthritis	-	1464	M05-M06	714
Macular degeneration	-	1528	H35.3	362.5
Osteoporosis	-	1309	M80-M81	733.0
Osteoarthritis	-	1465	M15-M19	715
**Clinical risk factors**	**Baseline measures (field ID)**	**Baseline verbal interview diagnosis codes**	**ICD-10 codes**	**ICD-9 codes**
Hypertension	High blood pressure diagnosis by physician (6150)<br>Taking medication for high blood pressure (6153, 6177)<br>Blood pressure SBP/DBP ≥ 140/90 mmHg (4079, 4080)	1065, 1072	I10-I15	401-405
Obesity	BMI ≥ 30 kg/m^2 (21001)	-	E66	278.0
Dyslipidemia	Taking cholesterol lowering medication (6153, 6177)<br>Blood cholesterol ≥ 240 / 38.67 (30690)<br>Blood LDL ≥ 160 / 38.67 (30780)<br>Blood HDL < 40 / 38.67 (30760)<br>Blood triglycerides ≥ 200 / 88.57 (30870)	-	E78	272

Verbal interview diagnosis codes are contained in the cancer (field ID 20001) and non-cancer illness (field ID 20002) variables. Field IDs for ICD variables: ICD-10 summary diagnoses (41270) and date of diagnosis (41280); ICD-9 summary diagnoses (41271) and date of diagnosis (41281). Incident disease cases were also identified using ICD-10 codes from cause of death information from linked death register data. Baseline prevalence for all diseases and clinical risk factors was calculated for all participants using baseline measures (including verbal interview diagnosis codes) + those with an ICD diagnosis before or on the date of recruitment into the UK Biobank. Incident cases are defined as those with an ICD date of diagnosis after the date of recruitment who do not have any prevalent diagnosis. Unless specific ICD subcategories are already given with dot separators, all ICD codes listed also include all subcategories (e.g., J44 includes J44, J44.0, J44.1, J44.8, J44.9). COPD: chronic obstructive pulmonary disease; BMI: body mass index: SBP: systolic blood pressure; DBP: diastolic blood pressure.

Supplementary Table 9. Components and calculation of the daily partial fiber score

Food variable<br>(UK Biobank field ID)	Portion specified in touchscreen question	Portion size	Estimated fiber content/portion (g)
Bread intake (1438)<br>Bread type (1448)	Slices	White bread: 36 g<br>Brown bread: 36 g<br>Wholemeal bread: 36 g<br>Other type of bread: 36 g	0.68<br>1.26<br>1.80<br>1.25
Breakfast cereal intake (1458)<br>Breakfast cereal type (1468)	Bowls	Bran cereal: 40 g<br>Biscuit cereal: 40 g<br>Oat cereal: 160 g<br>Muesli: 55 g<br>Other (e.g., cornflakes): 30 g	7.16<br>2.92<br>1.92<br>4.18<br>0.54

Estimated fiber content/portion take from Bradbury et al. (2018) [14].

Supplementary Table 10. Components and calculation of the LTPA and OPA scores

LTPA (field ID)	METs
Walking for pleasure (6164, 981, 971)	3.3 METs * mins/week
Strenuous sports (6164, 1001, 991)	8.0 METs * mins/week
Other exercises (6164, 3647, 3637)	4.5 METs * mins/week
Light DIY (6164, 1021, 1011)	2.25 METs * mins/week
Heavy DIY (6164, 2634, 2624)	4.5 METs * mins/week
**OPA (field ID)**	
Heavy physical work (816, 767)	4.5 METs * mins/week
Walking/standing work (806, 767)	2.25 METs * mins/week

LTPA: leisure-time physical activity; OPA: occupational physical activity; MET: metabolic equivalent of task; DIY: do-it-yourself;. METs used for each type of activity were taken from Pearce et al. (2020) [15].

Supplementary Table 11. Variables used for calculation of polygenic risk scores (PRS).

Outcome	UK Biobank derived PRS measure(s) used (field ID)
All-cause mortality	Bowel cancer (26218); breast cancer (26220); ovarian cancer (26232); prostate cancer (26267); type 2 diabetes (26285); cardiovascular disease (26223); coronary artery disease (26227); ischemic stroke (26248); Alzheimer's disease (26206); Parkinson's (26260); rheumatoid arthritis (26273); macular degeneration (26204); osteoporosis (26258); lung cancer; esophageal cancer; pancreatic cancer; leukemia; emphysema/COPD
Colorectal cancer	Bowel cancer (26218)
Lung cancer	(PGS000078) by Graff et al. (2021) [22]
Esophageal cancer	(PGS002298) by Choi et al. (2020) [23]
Liver cancer	None
Pancreatic cancer	(PGS000083) by Graff et al. (2021) [22]
Brain cancer	None
Leukemia	(PGS000077) by Graff et al. (2021) [22]
Lymphoma	None
Breast cancer	Breast cancer (26220)
Ovarian cancer	Ovarian cancer (26232)
Prostate cancer	Prostate cancer (26267)
Type 2 diabetes	Type 2 diabetes (26285)
Ischemic heart disease	Cardiovascular disease (26223); coronary artery disease (26227)
Cerebrovascular diseases	Ischemic stroke (26248)
Emphysema, COPD	(PGS001788) by Wang et al. (2021) [24]
Chronic liver diseases	Non-alcoholic fatty liver disease (PGS002282) by Schnurr et al. (2022) [25]<br>Liver cirrhosis (PGS000726) by Emdin et al. (2020) [26]
Chronic kidney diseases	(PGS000859) by Mansour Aly et al. (2021) [27]
All-cause dementia	Alzheimer's disease (26206)
Vascular dementia	Alzheimer's disease (26206)
Alzheimer's disease	Alzheimer's disease (26206)
Parkinson's disease and parkinsonism	Parkinson's (26260)
Rheumatoid arthritis	Rheumatoid arthritis (26273)
Macular degeneration	Macular degeneration (26204)
Osteoporosis	Osteoporosis (26258)
Osteoarthritis	Knee osteoarthritis (PGS002729) by Sedaghati-Khayat et al. (2022) [28]

Supplementary Table 12. Smoking associations with prostate cancer according to PSA test

	**No PSA test**		**Has PSA test**		**PSA test as covariate**	
	**Hazard Ratio [95% CI]**	**p-value**	**Hazard Ratio [95% CI]**	**p-value**	**Hazard Ratio [95% CI]**	**p-value**
Never smoker (reference)	-	-	-	-	-	-
Previous smoker	0.97 [0.91, 1.03]	0.31	0.93 [0.87, 1.00]	0.04	0.95 [0.91, 1.00]	0.04
Current smoker	0.90 [0.81, 0.99]	0.02	0.86 [0.75, 0.98]	0.02	0.88 [0.81, 0.95]	< 0.001

All models are Cox models with age as the timescale, stratified by 5-year birth cohorts, and with covariates for UK Biobank assessment center, household income, years of education, ethnicity, and IPAQ activity level. Sample sizes are n=137,598 for those with no PSA test, n=58,425 for those with a PSA test, and n=196,113 for final model not stratified by PSA test but including PSA test as a covariate.

**Supplementary Table 13.** Accelerometer vs. self-reported physical activity measures in relation to mortality				
	**Hazard Ratio [95% CI]**	**p-value**	**Hazard Ratio [95% CI]**	**p-value**
Overall acceleration average (milli-gravity)	0.95 [0.94, 0.95]	< 0.001		
IPAQ physical activity group			0.82 [0.80, 0.84]	< 0.001
Leisure time physical activity (LTPA)			0.85 [0.82, 0.88]	< 0.001
Occupational physical activity (OPA)			0.87 [0.84, 0.90]	< 0.001
Total sedentary time			1.10 [1.08, 1.13]	< 0.001
R^2	0.59		0.56	

Both models include covariates for age, sex, UK Biobank assessment center, household income, years of education, ethnicity, smoking status, and Townsend deprivation index.

Model	C-index	R^2
Model 2	0.73	0.50
Model 3	0.77	0.64
Model 4	0.77	0.65

Supplementary Table 14. Multivariable model metrics for chronic disease mortality in participants recruited in England (n=436,891). Each model was constructed as a Cox proportional hazards model. The outcome was mortality with any of the 25 chronic diseases studied in this paper listed as the primary or contributory cause. Model 2: age, sex, polygenic risk scores (PRS; including genetic principal components, and genotyping batch). Model 3: age, sex, exposome. Model 4: age, sex, exposome, PRS.

Model	C-index	R^2
Model 2	0.74	0.54
Model 3	0.79	0.70
Model 4	0.79	0.71

Supplementary Table 15. Multivariable model metrics for chronic disease mortality in participants recruited in Scotland/Wales. Each model was constructed as a Cox proportional hazards model. The outcome was mortality with any of the 25 chronic diseases studied in this paper listed as the primary or contributory cause. Model 2: age, sex, polygenic risk scores (PRS; including genetic principal components, and genotyping batch). Model 3: age, sex, exposome. Model 4: age, sex, exposome, PRS.

Supplementary Table 16. Performance of PRS and exposome + PRS models when including APOE and FOXO3 variants

a. Discovery set (recruited in England; n=436,891)		
**Model**	**C-index**	**R^2**
PRS only	0.71	0.46
Exposome + PRS	0.76	0.60
b. Validation set (recruited in Scotland/Wales; n=55,676)		
**Model**	**C-index**	**R^2**
PRS only	0.73	0.49
Exposome + PRS	0.77	0.66

Each model was constructed as a Cox proportional hazards model with all-cause mortality as the outcome. “PRS only” model includes age, sex, polygenic risk scores (PRS; including genetic principal components, and genotyping batch) for all other diseases studied, genotype for APOE status using rs429358 and rs7412, and a variant in FOXO3 previously associated with longevity (rs2802292). “Exposome + PRS” model includes all variables in the PRS only model + all 26 exposures associated with mortality. Results are shown both in models calculated in: (a) participants recruited in England (n=436,891); and (b) using the using linear predicted values from the models in (a) and outcome rates from the independent validation sample of participants recruited in Scotland/Wales (n=55,676).

Supplementary File legends

Supplementary File SF1. Data dictionary for all variables used in multiple imputation. Summary information about all baseline variables collected from the UK Biobank that were used in multiple imputation after variable exclusions. Information includes variable name used in analysis, UK Biobank field ID, original variable name in UK Biobank dataset provided to us, and URL link for each variable to the corresponding webpage on the UK Biobank showcase giving extensive detail for each variable.

Supplementary File SF2. Data dictionary for exposures used in XWAS analyses. Summary information about all exposome analyzed in the mortality XWAS, including the sex-specific reproduction factors analyzed in the sex-specific XWAS only. Information includes variable name used in analysis, UK Biobank field ID, original variable name in UK Biobank dataset provided to us, and URL link for each variable to the corresponding webpage on the UK Biobank showcase giving extensive detail for each variable.

Supplementary File SF3. Female XWAS output. We report all female-specific XWAS summary statistics. All effect estimates (hazard ratios, confidence intervals) shown are for analyses in the discovery set (n=118,815). FDR corrected p-values are given for both the discovery and replication analyses (FDR p-values will be NA for variables with an FDR p-value ≥ 0.05 in the discovery analysis, as these variables would not have been tested in the replication stage).

Supplementary File SF4. Male XWAS output. We report all male-specific XWAS summary statistics. All effect estimates (hazard ratios, confidence intervals) shown are for analyses in the discovery set (n=99,631). FDR corrected p-values are given for both the discovery and replication analyses (FDR p-values will be NA for variables with an FDR p-value ≥ 0.05 in the discovery analysis, as these variables would not have been tested in the replication stage).

Supplementary File SF5. Pooled XWAS output. We report all XWAS summary statistics from the final pooled XWAS. All effect estimates (hazard ratios, confidence intervals) shown are for analyses in the discovery set (n=218,446). FDR corrected p-values are given for both the discovery and replication analyses (FDR p-values will be NA for variables with an FDR p-value ≥ 0.05 in the discovery analysis, as these variables would not have been tested in the replication stage).

Supplementary File SF6. Disease interaction sensitivity output. We report all summary statistics from the disease sensitivity analysis conducted among UK Biobank participants where an interaction term was added between each exposure and a binary indicator of poor health at baseline. All effect estimates (hazard ratios, confidence intervals) shown are for analyses in the pooled dataset (n=436,891).

Supplementary File SF7. XWAS survival time exclusion sensitivity output. We report all summary statistics from the sensitivity analysis wherein we conducted a mortality XWAS excluding all UK Biobank participants who died within 4 years of baseline (n=431,394). All effect estimates (hazard ratios, confidence intervals) shown are for analyses in the pooled dataset.

Supplementary Files SF8. Proteomic aging analysis output. We report all summary statistics from the analysis testing associations between exposures still significant after XWAS and disease sensitivity analyses with plasma proteomic aging (n=45,441).

Supplementary Files SF9-SF33. Aging biomarker analysis output. We report all summary statistics from the aging biomarker analysis testing associations between 25 blood biomarkers and exposures still significant after cluster multivariable and disease sensitivity analyses (n=436,891).

Supplementary Files SF34-SF61. Incident disease and disease risk factor analysis output. We report all summary statistics from the incident chronic disease and clinical risk factor analysis testing associations between all 28 diseases/risk factors and exposures still significant after cluster multivariable and disease sensitivity analyses (n=436,891).

Supplementary File SF62. Data dictionary of phenotypes used in phenome-wide association study (PheWAS) analyses. Summary information about all phenotypes analyzed in per-exposure PheWAS. Information includes variable name used in analysis, UK Biobank field ID, original variable name in UK Biobank dataset provided to us, and URL link for each variable to the corresponding webpage on the UK Biobank showcase giving extensive detail for each variable.

Supplementary Files SF63-SF178. PheWAS output. We report all summary statistics from PheWAS testing associations between all exposures still significant after cluster multivariable and disease sensitivity analyses and all baseline phenotypes in the UK Biobank (n=436,891).

References

1. Bradbury, K.E., Murphy, N. & Key, T.J. Diet and colorectal cancer in UK Biobank: a prospective study. *Int J Epidemiol* **49**, 246-258 (2020).
2. Wood, A.M.*, et al.* Risk thresholds for alcohol consumption: combined analysis of individual-participant data for 599 912 current drinkers in 83 prospective studies. *Lancet* **391**, 1513-1523 (2018).
3. Fillmore, K., Stockwell, T., Chikritzhs, T., Bostrom, A. & Kerr, W. Moderate alcohol use and reduced mortality risk: systematic error in prospective studies and new hypotheses. *Ann Epidemiol* **17**, S16-23 (2007).
4. GBD 2016 Alcohol Collaborators. Alcohol use and burden for 195 countries and territories, 1990-2016: a systematic analysis for the Global Burden of Disease Study 2016. *Lancet* **392**, 1015-1035 (2018).
5. Fry, A.*, et al.* Comparison of Sociodemographic and Health-Related Characteristics of UK Biobank Participants With Those of the General Population. *Am J Epidemiol* **186**, 1026-1034 (2017).
6. Watson, N.F.*, et al.* Recommended Amount of Sleep for a Healthy Adult: A Joint Consensus Statement of the American Academy of Sleep Medicine and Sleep Research Society. *Sleep* **38**, 843-844 (2015).
7. Okbay, A.*, et al.* Genome-wide association study identifies 74 loci associated with educational attainment. *Nature* **533**, 539-542 (2016).
8. Lee, J.*, et al.* Gene discovery and polygenic prediction from a genome-wide association study of educational attainment in 1.1 million individuals. *Nat Genet* **50**, 1112-1121 (2018).
9. Mostafavi, H.*, et al.* Variable prediction accuracy of polygenic scores within an ancestry group. *eLife* **9**, e48376 (2020).
10. Nakamura, E. & Miyao, K. A Method for Identifying Biomarkers of Aging and Constructing an Index of Biological Age in Humans. *The Journals of Gerontology: Series A* **62**, 1096-1105 (2020).
11. Dockery, D.*, et al.* Distribution of forced expiratory volume in one second and forced vital capacity in healthy, white, adult never-smokers in six U.S. cities. *Am Rev Respir Dis* **131**, 511-520 (1985).
12. Chun, S., Kim, W. & Choi, K. Comparison between grip strength and grip strength divided by body weight in their relationship with metabolic syndrome and quality of life in the elderly. *PloS one* **14**, e0222040 (2019).
13. Garcia-Hermoso, A.*, et al.* Handgrip strength attenuates the adverse effects of overweight on cardiometabolic risk factors among collegiate students but not in individuals with higher fat levels. *Sci Rep* **9**, 6986 (2019).
14. Bradbury, K.E., Young, H.J., Guo, W. & Key, T.J. Dietary assessment in UK Biobank: an evaluation of the performance of the touchscreen dietary questionnaire. *J Nutr Sci* **7**, e6 (2018).
15. Pearce, M.*, et al.* Estimating physical activity from self-reported behaviours in large-scale population studies using network harmonisation: findings from UK Biobank and associations with disease outcomes. *Int J Behav Nutr Phys Act* **17**, 40 (2020).
16. Chudasama, Y.V.*, et al.* Physical activity, multimorbidity, and life expectancy: a UK Biobank longitudinal study. *BMC Med* **17**, 108 (2019).
17. Doherty, A.*, et al.* Large Scale Population Assessment of Physical Activity Using Wrist Worn Accelerometers: The UK Biobank Study. *PLoS One* **12**, e0169649 (2017).

18. Larsson, S.C., *et al.* Smoking, alcohol consumption, and cancer: A mendelian randomisation study in UK Biobank and international genetic consortia participants. *PLoS Med* **17**, e1003178 (2020).
19. Rohrmann, S., *et al.* Smoking and the risk of prostate cancer in the European Prospective Investigation into Cancer and Nutrition. *Br J Cancer* **108**, 708-714 (2013).
20. Watters, J.L., Park, Y., Hollenbeck, A., Schatzkin, A. & Albanes, D. Cigarette smoking and prostate cancer in a prospective US cohort study. *Cancer Epidemiol Biomarkers Prev* **18**, 2427-2435 (2009).
21. Watts, E.L., *et al.* Circulating insulin-like growth factors and risks of overall, aggressive and early-onset prostate cancer: a collaborative analysis of 20 prospective studies and Mendelian randomization analysis. *Int J Epidemiol* (2022).
22. Graff, R.E., *et al.* Cross-cancer evaluation of polygenic risk scores for 16 cancer types in two large cohorts. *Nat Commun* **12**, 970 (2021).
23. Choi, J., Jia, G., Wen, W., Long, J. & Zheng, W. Evaluating polygenic risk scores in assessing risk of nine solid and hematologic cancers in European descendants. *Int J Cancer* **147**, 3416-3423 (2020).
24. Wang, Y., *et al.* Global biobank analyses provide lessons for developing polygenic risk scores across diverse cohorts. *medRxiv* (2021).
25. Schnurr, T.M., *et al.* Interactions of physical activity, muscular fitness, adiposity, and genetic risk for NAFLD. *Hepatol Commun* **6**, 1516-1526 (2022).
26. Emdin, C.A., *et al.* Association of Genetic Variation With Cirrhosis: A Multi-Trait Genome-Wide Association and Gene-Environment Interaction Study. *Gastroenterology* **160**, 1620-1633.e1613 (2021).
27. Mansour Aly, D., *et al.* Genome-wide association analyses highlight etiological differences underlying newly defined subtypes of diabetes. *Nat Genet* **53**, 1534-1542 (2021).
28. Sedaghati-Khayat, B., *et al.* Risk Assessment for Hip and Knee Osteoarthritis Using Polygenic Risk Scores. *Arthritis Rheumatol* **74**, 1488-1496 (2022).
