# Supplementary material for: Integrating the environmental and genetic architectures of aging and mortality
Source: Nat Med. 2025 Feb 19;31(3):1016–25. doi: 10.1038/s41591-024-03483-9 (PMC11922759; doi:10.1038/s41591-024-03483-9)
Supplement: Supplementary file 2 — Reporting Summary [file 41591_2024_3483_MOESM2_ESM.pdf]

nature portfolio

Corresponding author(s): Austin Argentieri

Last updated by author(s): Dec 3, 2024

Reporting Summary

Nature Portfolio wishes to improve the reproducibility of the work that we publish. This form provides structure for consistency and transparency in reporting. For further information on Nature Portfolio policies, see our Editorial Policies and the Editorial Policy Checklist.

Statistics

For all statistical analyses, confirm that the following items are present in the figure legend, table legend, main text, or Methods section.

n/a	Confirmed	
☐	☒	The exact sample size (*n*) for each experimental group/condition, given as a discrete number and unit of measurement
☐	☒	A statement on whether measurements were taken from distinct samples or whether the same sample was measured repeatedly
☐	☒	The statistical test(s) used AND whether they are one- or two-sided<br>*Only common tests should be described solely by name; describe more complex techniques in the Methods section.*
☐	☒	A description of all covariates tested
☐	☒	A description of any assumptions or corrections, such as tests of normality and adjustment for multiple comparisons
☐	☒	A full description of the statistical parameters including central tendency (e.g. means) or other basic estimates (e.g. regression coefficient) AND variation (e.g. standard deviation) or associated estimates of uncertainty (e.g. confidence intervals)
☐	☒	For null hypothesis testing, the test statistic (e.g. *F*, *t*, *r*) with confidence intervals, effect sizes, degrees of freedom and *P* value noted<br>*Give P values as exact values whenever suitable.*
☒	☐	For Bayesian analysis, information on the choice of priors and Markov chain Monte Carlo settings
☐	☒	For hierarchical and complex designs, identification of the appropriate level for tests and full reporting of outcomes
☐	☒	Estimates of effect sizes (e.g. Cohen's *d*, Pearson's *r*), indicating how they were calculated

Our web collection on statistics for biologists contains articles on many of the points above.

Software and code

Policy information about availability of computer code

Data collection	No software was used.
Data analysis	R version 4.2.2 and PLINK version 2.0.

For manuscripts utilizing custom algorithms or software that are central to the research but not yet described in published literature, software must be made available to editors and reviewers. We strongly encourage code deposition in a community repository (e.g. GitHub). See the Nature Portfolio guidelines for submitting code & software for further information.

Data

Policy information about availability of data

All manuscripts must include a data availability statement. This statement should provide the following information, where applicable:

- Accession codes, unique identifiers, or web links for publicly available datasets
- A description of any restrictions on data availability
- For clinical datasets or third party data, please ensure that the statement adheres to our policy

UK Biobank data are available through a procedure described at https://www.ukbiobank.ac.uk/enable-your-research. Summary statistics from all analysis stages are included in Supplementary Files SF3-SF178. All polygenic risk score summary statistics taken from the Polygenic Score Catalog (PGS) are publicly available at https://www.pgscatalog.org/.

Research involving human participants, their data, or biological material

Policy information about studies with human participants or human data. See also policy information about sex, gender (identity/presentation), and sexual orientation and race, ethnicity and racism.

Reporting on sex and gender	Initial exposome-wide association study (XWAS) analyses were carried out separately by sex to test for differences in associations by sex. After finding a strong correlation between the betas in the sex-specific XWAS, we pooled both sexes together and conducted a single XWAS. All subsequent Cox models included a strata term for sex, and all subsequent linear and logistic regression models included a covariate for sex. Descriptive statistics in Fig. 2 are show according to sex.
Reporting on race, ethnicity, or other socially relevant groupings	Self-reported ethnicity was used as a covariate in all models.
Population characteristics	The final study sample included 492,567 UK Biobank participants (Fig. 1). All analyses were carried out using UK Biobank participants recruited in England (n=436,891). Participants recruited in Scotland/Wales (n=55,676) were held out as a validation set used only to validate final multivariable disease models. There were 31,716 deaths from all causes among participants recruited in England after a median 12.5 years of follow up (Table S1). The majority (74.5%) of deaths were premature deaths (i.e., occurring before 75 years of age; Fig. 2a) and 75% of deaths occurred in those who were overweight or obese with a body mass index (BMI) ≥ 25 kg/m2 (Fig. 2b). Women had a lower all-cause mortality rate compared with men (5.4% in women vs 9.4% in men; Table S1). Compared with men, more women reported being never smokers, reported lower levels of income, and reported less years of education (Fig. 2).
Recruitment	Participants were recruited to the UK Biobank between 2006-2010. Further information on recruitment has been published previously (https://www.ukbiobank.ac.uk/media/gnkeyh2q/study-rationale.pdf).
Ethics oversight	UK Biobank data use (Project Application Number 61054) was approved by the UK Biobank according to their established access procedures. UK Biobank has approval from the North West Multi-centre Research Ethics Committee (MREC) as a Research Tissue Bank (RTB), and as such researchers using UK Biobank data do not require separate ethical clearance and can operate under the RTB approval.

Note that full information on the approval of the study protocol must also be provided in the manuscript.

Field-specific reporting

Please select the one below that is the best fit for your research. If you are not sure, read the appropriate sections before making your selection.

☐ Life sciences ☒ Behavioural & social sciences ☐ Ecological, evolutionary & environmental sciences

For a reference copy of the document with all sections, see nature.com/documents/nr-reporting-summary-flat.pdf

Behavioural & social sciences study design

All studies must disclose on these points even when the disclosure is negative.

Study description	The study used quantitative methods within the context of a prospective cohort study.
Research sample	Our study uses secondary data from 492,567 UK Biobank (UKB, 54% female, age range: 40-71 years) participants. We chose the UK Biobank for the breadth of exposures, phenotypes, and biological data available, allowing for comprehensive and integrative modeling. We used previously collected self-report questionnaire data, data from clinical interviews, genotyping data from blood samples collected at baseline, biochemical measures from blood samples collected at baseline, and hospital diagnosis and mortality information from linked inpatient and mortality register data.
Sampling strategy	The final study sample included 492,567 UK Biobank participants. All analyses were carried out using UK Biobank participants recruited in England (n=436,891). Participants recruited in Scotland/Wales (n=55,676) were held out as a validation set used only to validate final multivariable disease models. Our large dataset makes it one of the largest exposome-wide studies carried out to date, and the UK Biobank resource also allows for a much broader diversity of exposures to be tested than those that are routinely tested in XWAS. Power analyses were not conducted for this study - we used all participants with data available in the UKB.
Data collection	Participants were recruited to the UKB between 2006-2010. Further information on UKB recruitment and data collection has been published previously (https://www.ukbiobank.ac.uk/media/gnkeyh2q/study-rationale.pdf). No new data were collected from UKBparticipants for this study. Researchers in our study were not blinded to the study hypothesis.
Timing	Baseline UKB collection took place from March 15 2006 until September 27 2010. Follow up mortality and incident disease data were collected until October 31 2021, leaving 10-15 years of follow up.
Data exclusions	We considered the entire UK Biobank cohort for inclusion in our study and only excluded n=9,835 who were adopted (to maintain consistency in exposures collected across all participants), n=6 who were aged less than 40 years at baseline, n=2 without valid ICD diagnosis data, and n=95 participants who requested to be removed from the UK Biobank.

Non-participation	n=95 participants requested to be removed from the UK Biobank during the course of our study. Their reasons for wanting to be removed were not reported to investigators by the cohort.
Randomization	Participants were randomly assigned to discovery and replication groups for the XWAS. Selection of participants to hold out as a validation set was not done randomly but done according to where participants were recruited to the UK Biobank. All other analyses were conducted in the full sample of UK Biobank participants recruited in England.

Reporting for specific materials, systems and methods

We require information from authors about some types of materials, experimental systems and methods used in many studies. Here, indicate whether each material, system or method listed is relevant to your study. If you are not sure if a list item applies to your research, read the appropriate section before selecting a response.

Materials & experimental systems

n/a	Involved in the study
☒	☐ Antibodies
☒	☐ Eukaryotic cell lines
☒	☐ Palaeontology and archaeology
☒	☐ Animals and other organisms
☒	☐ Clinical data
☒	☐ Dual use research of concern
☒	☐ Plants

Methods

n/a	Involved in the study
☒	☐ ChIP-seq
☒	☐ Flow cytometry
☒	☐ MRI-based neuroimaging
